# Supplementary material for: Boron-assisted synthesis of compositionally complex amorphous oxides via short-range-order-constrained generative design
Source: Sci Adv. 2026 Sep 25;12(39):eaef4658. doi: 10.1126/sciadv.aef4658 (PMC13614375; doi:10.1126/sciadv.aef4658)
Supplement: Supplementary file 1 [file sciadv.aef4658_sm.pdf]

Science Advances
AAAS

Supplementary Materials for

Boron-assisted synthesis of compositionally complex amorphous oxides via short-range-order-constrained generative design

Honglin Li *et al.*

Corresponding author: Chuhao Liu, chuhao_liu@fzu.edu.cn; Mufan Li, mufanli@pku.edu.cn; Yanchao Wang, wyc@calypso.cn; Wan-Lu Li, wal019@ucsd.edu

Sci. Adv. **12**, eaef4658 (2026)
DOI: 10.1126/sciadv.aef4658

This PDF file includes:

Supplementary Text
Supplementary Algorithm 1
Tables S1 to S8
Figs. S1 to S16
References

S1 Supplementary Text

A. Algorithm for PDM Calculation

Below, we present the pseudocode for computing the PDM.

Supplementary Algorithm 1. PDM calculation

Require: atomic positions $X \in \mathbb{R}^{N\times 3}$; atomic species labels $\mathcal{S} = \{s_1, \ldots, s_N\}$; lattice matrix $L \in \mathbb{R}^{3\times 3}$; cutoff $r_c = 5.0\,\text{Å}$
Ensure: unordered-pair count map $\text{PDM}\{\alpha\beta \to n\}$

initialize $D \leftarrow \mathbf{0}^{N\times N}$
for $i \leftarrow 1$ to N **do**
  for $j \leftarrow 1$ to N **do**
    if $i \neq j$ **then**
      $\boldsymbol{\delta} \leftarrow X[j] - X[i] - \text{round}\big((X[j] - X[i])L^{-1}\big)L$
      $D[i,j] \leftarrow \|\boldsymbol{\delta}\|$
    end if
  end for
end for
initialize $\text{PDM}[\alpha\beta] \leftarrow 0$ for all unordered species pairs (α, β) with $\alpha \leq \beta$
for $i \leftarrow 1$ to $N-1$ **do**
  for $j \leftarrow i+1$ to N **do**
    if $D[i,j] < r_c$ **then**
      $\text{key} \leftarrow \textsc{Sort}(s_i, s_j)$
      $\text{PDM}[\text{key}] \leftarrow \text{PDM}[\text{key}] + 1$
    end if
  end for
end for

Supplementary Algorithm 1 provides a direct computational recipe for the PDM defined in Equation 1. In a single pass, it first builds the full pairwise distance matrix using raw Euclidean norms and the minimum-image convention under periodic boundary conditions, so that each entry D_{ij} correctly reflects the shortest separation between atoms i and j. It then initializes an empty dictionary for PDM counts and loops over every unordered atom pair $(i < j)$. Whenever $D_{ij} < r_c$, the species labels (s_i, s_j) are sorted into a key (e.g. "AB"), and the corresponding count is incremented by one. The final dictionary therefore contains the near-neighbour counts for all element pairs, which are then assembled into the $K \times K$ PDM matrix used as the Cond-CDVAE conditioning descriptor.

To illustrate the construction of the Pair-Density Matrix (PDM), we consider a minimal four-atom *cubic* cell with lattice constant $a = 3.0\,\text{Å}$. Two A atoms are placed at $(0,0,0)$ and $(1,0,0)$, and two B atoms are placed at $(0,1,0)$ and $(1,1,0)$ (Cartesian coordinates, in Å throughout). Periodic boundary conditions (PBC) are applied; because all pairwise separations are smaller than $a/2$, no minimum-image wrapping occurs. For clarity, we use a demonstration cutoff of $r_c^{\text{demo}} = 1.1\,\text{Å}$ (the production value elsewhere in the paper is $5.0\,\text{Å}$).

Exactly four unordered pairs—$(1,2)$, $(1,3)$, $(2,4)$, and $(3,4)$—lie within the cutoff. Applying the sorting-and-counting rule in Algorithm 1 gives

$$\text{PDM}_{AA} = 1, \qquad \text{PDM}_{AB} = 2, \qquad \text{PDM}_{BB} = 1,$$

so that, in the basis $\{A, B\}$,

$$\text{PDM}\big(r_c^{\text{demo}}\big) = \begin{pmatrix} 1 & 2 \\ 2 & 1 \end{pmatrix}.$$

The off-diagonal entry counts every distinct A–B neighbour pair, and the symmetry $\text{PDM}_{\alpha\beta} = \text{PDM}_{\beta\alpha}$ is enforced by construction. Running the same coordinates through our public script `compute_pdm.py` returns the same dictionary `{AA: 1, AB: 2, BB: 1}`, thereby validating Equation 1 and Algorithm 1.

TABLE S1. Inter-atomic distances in the 4-atom cell. An asterisk (*) marks pairs that satisfy $d_{ij} < r_c^{\mathrm{demo}}$.

i	j	d_{ij} (Å)	species pair
1	2	1.000*	A–A
1	3	1.000*	A–B
1	4	1.414	A–B
2	3	1.414	A–B
2	4	1.000*	A–B
3	4	1.000*	B–B

B. Evaluation of the Generative Model

To evaluate the effectiveness of ApolloX in modeling complex disordered systems, we applied it to a representative multicomponent composition, $Fe_{12}Co_{12}Ni_{12}Mo_{12}B_{12}O_{60}$ (120 atoms). To maintain charge neutrality while preserving an equimolar ratio among the metallic species and boron, we adopted a Fe:Co:Ni:Mo:B:O atomic ratio of 12:12:12:12:12:60. This chemically diverse, boron-rich oxide provides a stringent test bed for benchmarking ApolloX in predicting thermodynamic stability, short-range order, and structure–property relationships in amorphous or metastable materials.

A total of 10,000 candidate $Fe_{12}Co_{12}Ni_{12}Mo_{12}B_{12}O_{60}$ structures were generated by random elemental substitution on a BCC parent phase within a 120-atom cell. Analogous energy-distribution tests were also performed for FCC and HCP configurations, as discussed in Supplementary Section C. These structures constitute the initial dataset for training and evaluating the generative model.

The pair-density matrices (PDMs) of these structures were then used to train the conditional generative model (Cond-CDVAE). To assess model accuracy, we selected a reference PDM as the target and generated 100 structures. As shown in Fig. S1a, the average relative pairwise deviations between the generated and target PDMs are below 25% for most element pairs.

We further evaluated the reconstruction accuracy on a test set of 1,000 structures. For each target PDM, one structure was generated, and the mean relative PDM error was computed by averaging over all matrix elements. As summarized in Fig. S1b, 90% of the generated structures exhibit mean relative errors below 20%, indicating that the generative model accurately captures the targeted CSRO characteristics. Novelty was assessed by comparing the 1,000 generated structures against the 10,000 structures in the training set. The novelty fraction, defined as the proportion of generated structures with no matches in the training data, decreases systematically with increasing similarity threshold δ, as shown in the inset of Fig. S1b, because looser matching criteria classify more generated structures as non-novel. Structural diversity was further quantified using uniqueness, coverage recall, and coverage precision as functions of δ. As shown in Fig. S1c, uniqueness decreases monotonically with increasing δ, whereas both coverage recall and coverage precision increase, revealing a clear trade-off between structural distinctiveness and coverage of the target structural space.

To demonstrate the capability of ApolloX for amorphous structure search, we initialized a PSO-based structural evolution with 100 Cond-CDVAE-generated structures as the initial population and evolved them over 15 generations. The evolution of the lowest-energy structure across generations is shown in Supplementary Fig. S1d, with the thermodynamically most favorable configuration shown in the inset. The lowest-energy amorphous $Fe_{12}Co_{12}Ni_{12}Mo_{12}B_{12}O_{60}$ structure, with an energy of -1045.39 eV, emerged by the third generation, highlighting the efficiency of the PSO algorithm in optimizing PDM-constrained energy landscapes.

To further elucidate the mapping between chemical short-range order (CSRO) and the potential-energy landscape, we generated a statistical ensemble of 2,400 structures. After geometric optimization, the PDM of the thermodynamically most stable configuration (i.e., the lowest-energy structure) was extracted as the reference ground-state motif. The ensemble was then stratified into four groups according to the magnitude of the PDM deviation from this reference, defined as the accumulated absolute difference of matrix elements.

As illustrated in Supplementary Fig. S1e, a clear correlation is observed between CSRO fidelity and energetic stability: structures with PDMs most similar to the ground state (Group 1) are concentrated in deep, low-energy basins, whereas those with larger PDM deviations (Groups 2–4) show a systematic shift toward higher-energy, less stable regions. This monotonic broadening and upward shift of the energy distribution confirm that the PDM descriptor captures essential energetic information of the amorphous network, thereby supporting its validity as a structural constraint for targeting thermodynamically favorable phases in the generative model. Fig. S1f presents the five representative low-energy structures identified by ApolloX together with their corresponding PDM maps. As the structural energy increases from S_1 to S_5, the associated PDM patterns evolve systematically, indicating that distinct energy levels are accompanied by different short-range-ordering characteristics. In particular, the changes in the intensity distribution and local pairwise features of the PDM reveal that structures located at different positions of the energy landscape possess distinguishable CSRO motifs. This result demonstrates that the PDM descriptor is not only capable of encoding short-range order, but also sensitive enough to resolve structurally meaningful differences across different energy regimes, thereby providing an effective coordinate for identifying and distinguishing amorphous configurations at different stability levels.

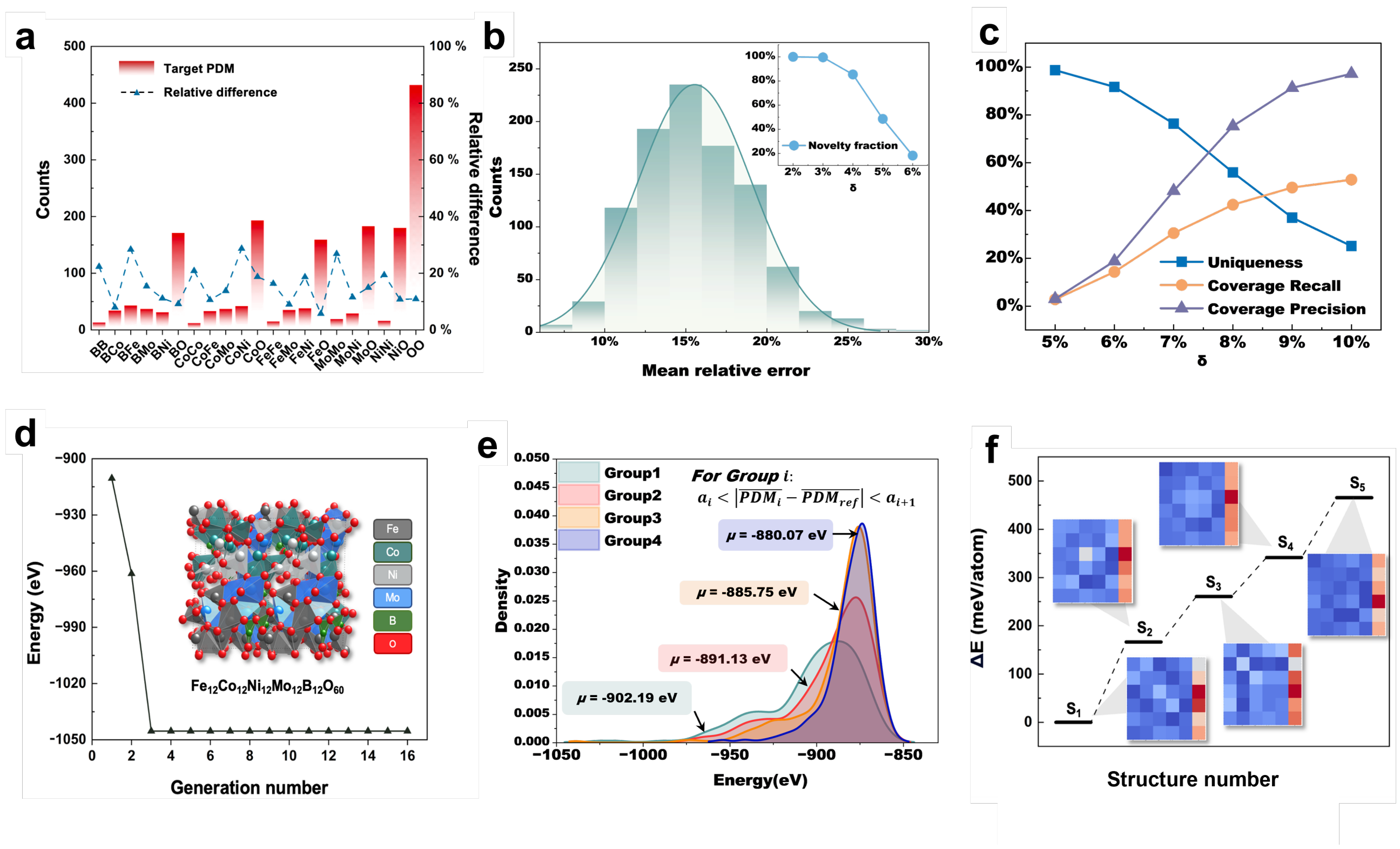

FIG. S1. **Model prediction of structural energy distribution and analysis.** (a) Target pair-distribution matrix (PDM) and the relative deviation of generated structures, demonstrating the model's ability to capture short-range ordering with high fidelity. (b) Histogram of the mean relative PDM errors. Inset: novelty fraction plotted as a function of the similarity threshold δ. (c) Diversity metrics–uniqueness, coverage recall, and coverage precision–plotted as functions of the similarity threshold δ. (d) Particle-swarm optimisation (PSO trajectory; the minimum-energy structure appears in the third generation, and one representative thermodynamically stable structure is shown. (e) Energy distributions of structures stratifed by increasing PDM deviation from the lowest-energy configuration (Group 1: smallest deviation;Group 4: largest deviation), demonstrating the correlation between PDM fidelity and thermodynamic stability. (f) The five lowest-energy structures obtained with ApolloX; their PDMs (inset) exhibit a characteristic short-range-ordering motif.

TABLE S2. Comparison of configurational energy calculations for different methods.

Method	Input information	$E_{\min}$ (eV)	$p(E < -960\text{ eV})$ (%)	E_{ave} (eV)
Random	Composition + parent phase	-970.0	0.3	-882.9
SQS	Composition + parent phase	-974.2	0.12	-882.4
MC	Composition + parent phase	-923.8	none	-873.3
MMC	Composition + lower energy	-979.9	1.2	-906.9
ApolloX	Composition + parent phase	-1045.45	1.7	-888.21

Note: The PSO algorithm (composition + symmetry) failed to generate structures, and the CE + MC method (lattice sites + relaxed energies) did not converge.

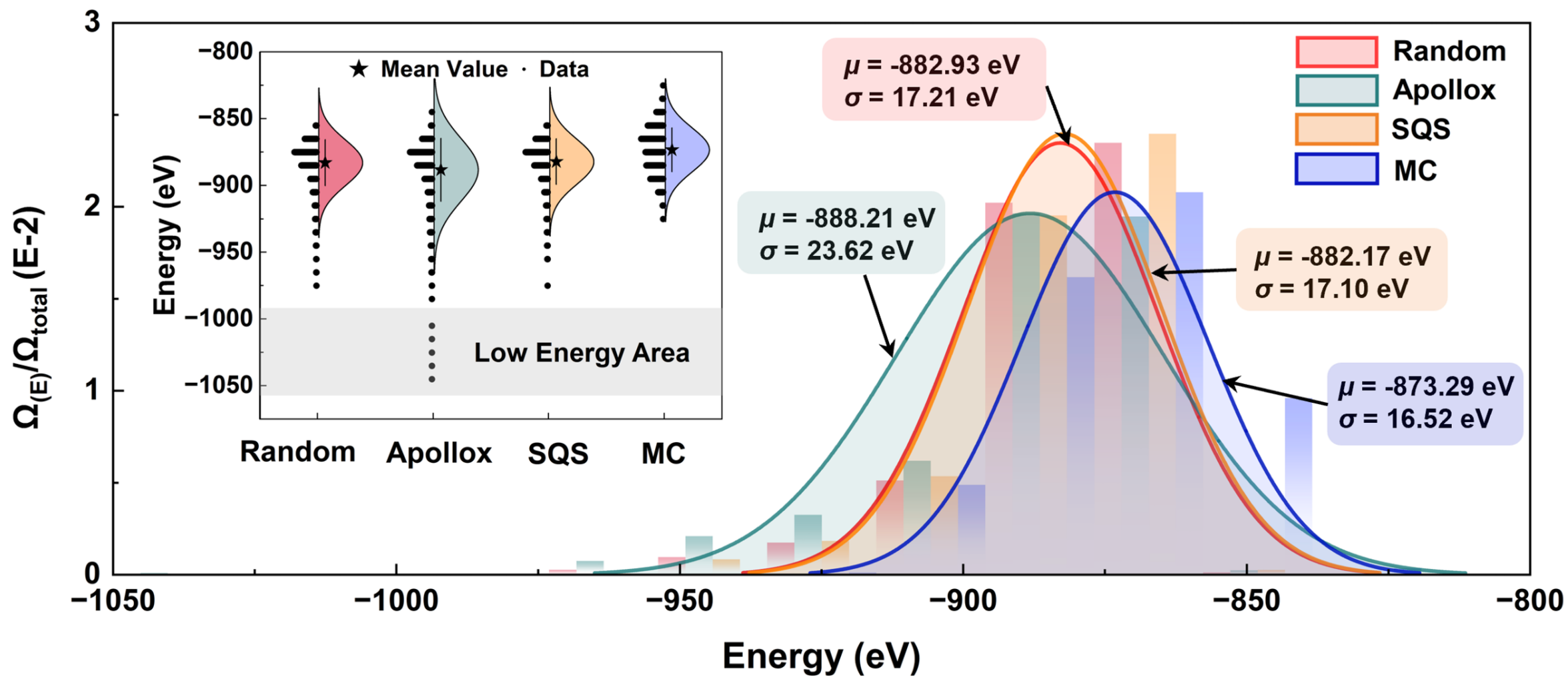

FIG. S2. Energy distributions of structures produced by the Random, ApolloX, SQS, and MC methods. The bar chart highlights the greater abundance of ApolloX structures in the low-energy regime. Inset: violin plots showing the global mean energies and the energy distributions within the window from −1050 to −990 eV.

TABLE S3. **Hyperparameters of Cond-CDVAE model used in this work.** Each element type is embedded by a vector of length 50. $\mathrm{MLP}_{\mathrm{zc}}$ is a multi-layer perceptron for concatenated latent vector and condition embedding vector. DimeNet++ and GemNet-dQ are used as $\mathrm{PGNN}_{\mathrm{Enc}}$ and $\mathrm{PGNN}_{\mathrm{Dec}}$, respectively.

**Model**	**Value**
Element type embedding	50
vectorized PDM dimension	197
$\mathrm{MLP}_{\mathrm{zc}}$ number of layers	3
$\mathrm{MLP}_{\mathrm{zc}}$ number of hidden channels	64
$\mathrm{MLP}_{\mathrm{L}}$ number of layers	1
$\mathrm{MLP}_{\mathrm{L}}$ number of hidden channels	256
$\mathrm{PGNN}_{\mathrm{Enc}}$ number of blocks	4
$\mathrm{PGNN}_{\mathrm{Enc}}$ number of hidden channels	128
$\mathrm{PGNN}_{\mathrm{Enc}}$ interaction embedding size	128
$\mathrm{PGNN}_{\mathrm{Dec}}$ number of blocks	4
$\mathrm{PGNN}_{\mathrm{Dec}}$ number ofhidden channels	128
Loss weight $\lambda_{\mathbf{L}}$	10
Loss weight $\lambda_{\mathbf{X}}$	10
Loss weight β	0.01
**Optimizer**	**Value**
Optimizer type	Adam
Learning rate	1e-4
Learning rate scheduler	ReduceLROnPlateau
Scheduler patience (epoch)	30
Scheduler factor (epoch)	0.6
Minimal learning rate	1e-5
**Data**	**Value**
Batch size	128

C. Comparative Energy Evaluation of BCC, FCC, and HCP Structures

We conducted energy distribution calculations on 1,500 structures for each of the BCC, FCC, and HCP configurations to ensure a comprehensive comparison. The energy distributions of these configurations are shown in Fig. S3. The results clearly demonstrate that the BCC lattice consistently outperforms FCC and HCP in terms of stability. Specifically, the average energy of the BCC system (-7.50 eV/atom) is lower than that of FCC (-7.46 eV/atom) and slightly lower than HCP (-7.34 eV/atom). The minimum energy structure in the BCC system also exhibits the lowest energy (-9.05 eV/atom) among the three lattices, indicating its superior potential to form highly stable configurations. These findings strongly support the rationality of our choice to use the BCC lattice.

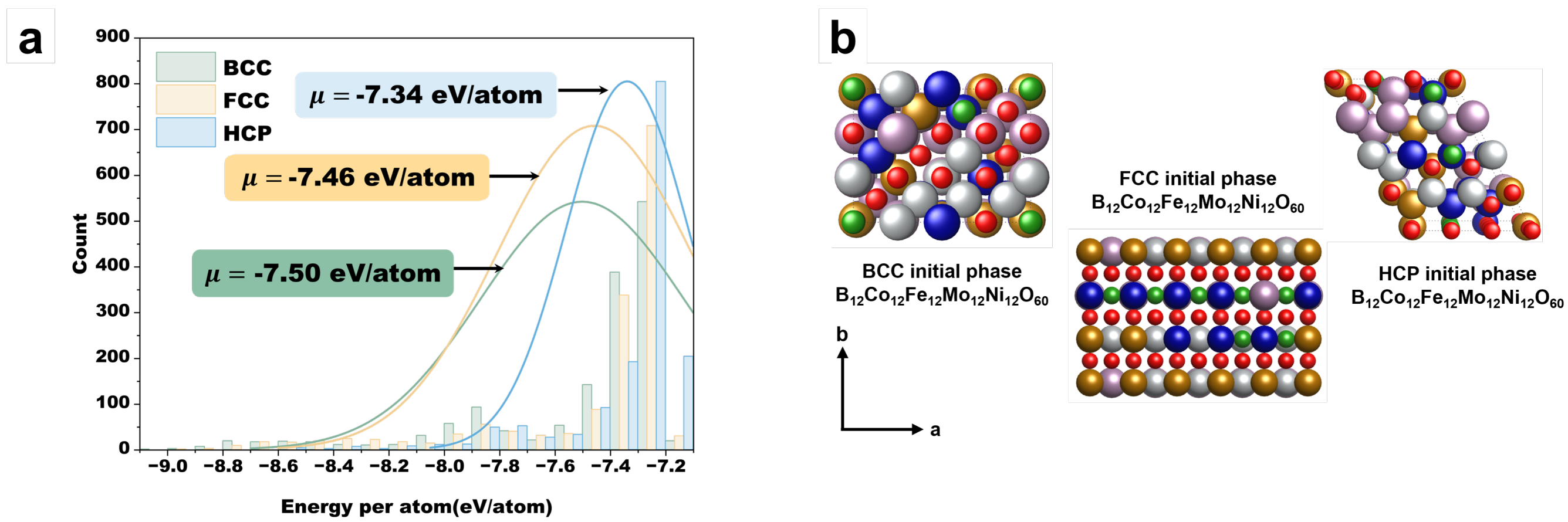

FIG. S3. **Evaluation of structural energy landscapes for different prototype configurations.** (a) Energy distributions of 1500 generated structures initialized from three distinct crystal prototypes: body-centered cubic (BCC), face-centered cubic (FCC), and hexagonal close-packed (HCP). The BCC-initialized structures exhibit a broader distribution and lower average energy, suggesting better thermodynamic stability. (b) Visual representations of the three initial crystal prototype phases used for structure generation: body-centered cubic (BCC), face-centered cubic (FCC), and hexagonal close-packed (HCP) configurations for the B12Co12Fe12Mo12Ni12O60 composition.

D. Validation of DPA-2 Energy Accuracy Against DFT Calculations

To evaluate the predictive accuracy of the DPA-2 machine-learned potential, we compared its energy estimations against density functional theory (DFT) calculations across a set of representative structures. The energy discrepancies, defined as $\Delta E_{\mathrm{DFT-DPA2}}$, are summarized in Fig. S4. Overall, the majority of structures exhibit excellent agreement between DPA-2 and DFT, with absolute energy differences falling within ± 5 meV/atom. This level of accuracy is comparable to typical thresholds reported for high-quality machine-learned interatomic potentials.

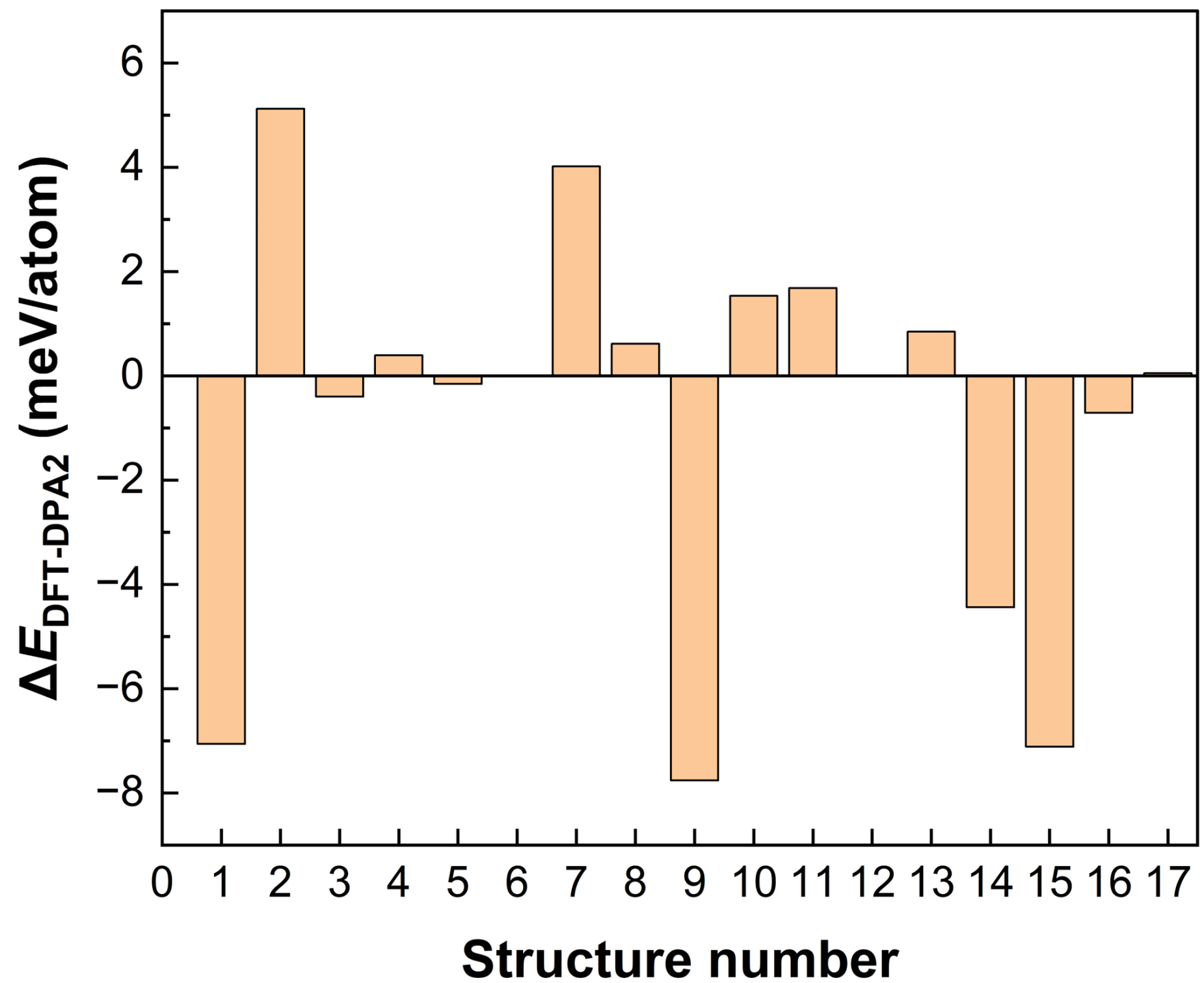

FIG. S4. **Accuracy analysis of DPA-2.** Compared the energy differences between DFT and DPA-2 for structures within different energy intervals to evaluate the accuracy of the MLP. The horizontal axis represents the number of structures, while the vertical axis shows the energy discrepancy between DFT and DPA-2 for each respective structure.

E. Molecular dynamics method and diffusion rate analysis

AIMD calculations for the representative Group 1–3 systems were performed with VASP using the PBE functional and PAW potentials. These calculations provided reference configurations for structural analysis and DPA-2 fine-tuning and validation. For the expanded series containing 0–20 at.% B in increments of approximately 2 at.%, ten independent DPA-2 trajectories were performed for each composition. Each trajectory was equilibrated at 300 K for 10 ps and then linearly heated to 2050 K over 24 ps. Species-resolved MSD and short-range-order descriptors were averaged over the ten trajectories. Because the simulations were non-isothermal, the MSD results represent atomic mobility during heating rather than equilibrium diffusion at a fixed temperature.

The diffusion rate (D) can be inferred from the slope of the MSD curve, which can be calculated as:

$$\langle (r(t) - r(0))^2 \rangle = 6Dt \tag{S1}$$

As the boron content increases, the overall diffusion rate of the system decreases, as shown in Fig. S5a. This trend is also observed for metal and boron species (Fig. 3C in the main text). Additionally, oxygen atoms exhibit higher diffusion rates compared to metals and boron, with boron being the most sluggish component. Consequently, boron plays a key role in determining the crystallization rate, which in turn influences the degree of amorphization.

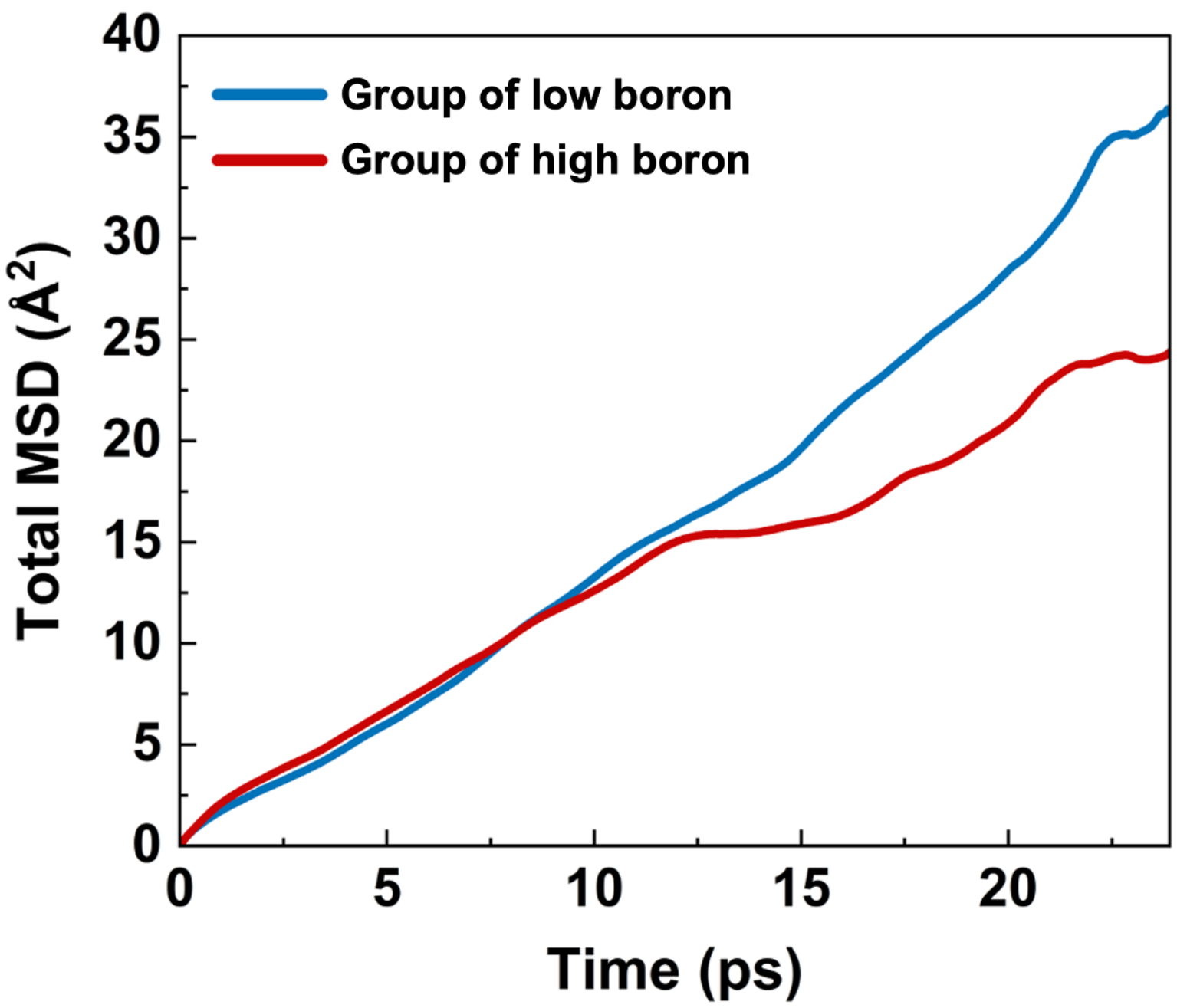

FIG. S5. **Diffusion rate analysis across groups with different boron contents.** Diffusion coefficients for all atoms. Molecular dynamics simulations were performed under a slow heating ramp from 300 K to 2050 K over a total duration of 24 ps.

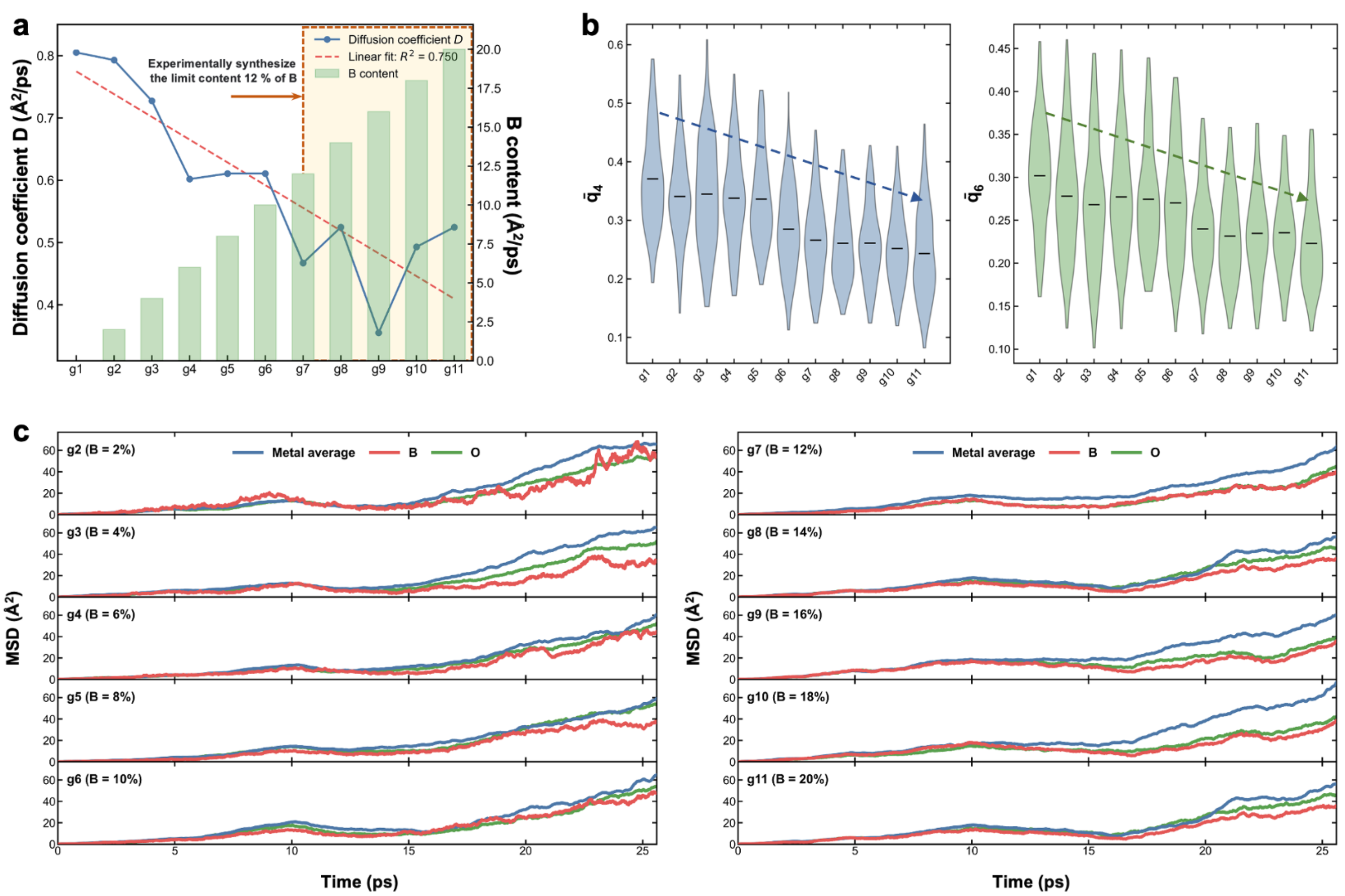

FIG. S6. **Boron-dependent diffusion suppression and disorder evolution in the theoretical** $g1$–$g11$ **series.** (a) Diffusion coefficients extracted from MD simulations as a function of composition, where the $g1$–$g11$ series corresponds to increasing boron content from 0% to 20% in 2% increments. The green bars indicate the nominal B content, and the blue line denotes the calculated diffusion coefficient D. The red dashed line shows the linear fit, revealing an overall decrease in diffusivity with increasing boron concentration. The shaded region and annotation mark the experimentally accessible synthesis window up to 12% B. (b) Violin plots of the bond-orientational order parameters $\bar{q}_4$ and $\bar{q}_6$ for the $g1$–$g11$ series. The dashed guide lines indicate a systematic decrease in both order parameters with increasing boron content, consistent with enhanced structural disorder and progressive amorphization. (c) Time evolution of the mean squared displacement (MSD) for the metal average, B, and O atoms in the $g2$–$g11$ structures. Increasing boron content leads to an overall suppression of atomic mobility, with boron exhibiting the slowest diffusion. In the boron-rich systems, the MSD curves show a more pronounced stagnation regime, indicating restricted atomic rearrangement and supporting the role of boron in stabilizing amorphous local environments.

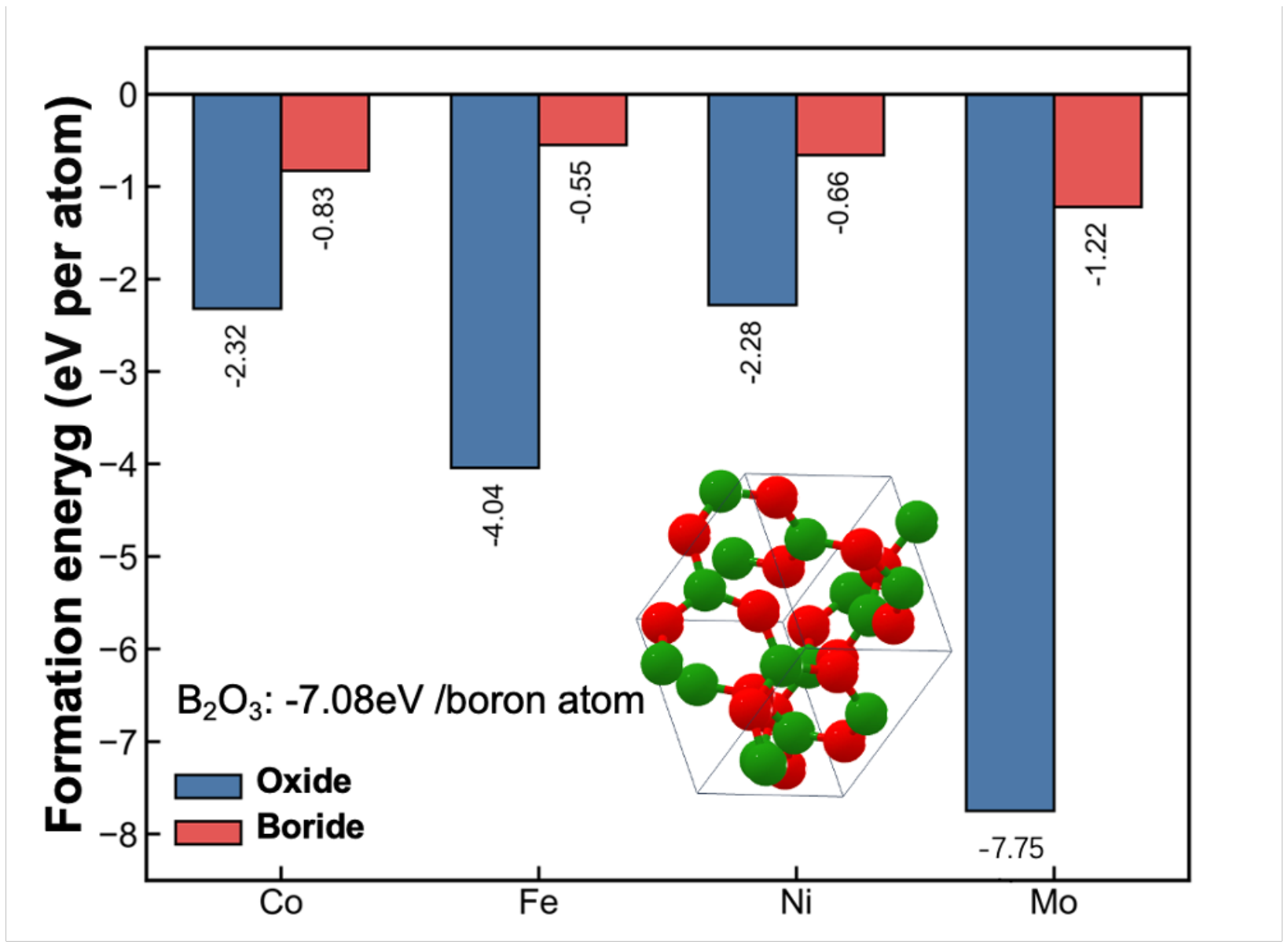

FIG. S7. **Comparison of oxide and boride formation energies for Co, Fe, Ni, and Mo, with B_2O_3 as a reference.** The calculated formation energies per atom of the corresponding oxides (blue) and borides (red) are compared for Co, Fe, Ni, and Mo. For all four metals, the oxide phases are energetically more favorable than the corresponding borides, indicating a stronger thermodynamic preference for metal–oxygen bonding over metal–boron bonding under oxygen-rich conditions. The annotated value for B_2O_3 (-7.08 eV per boron atom) further highlights the exceptional thermodynamic stability of boron oxide. The inset shows the crystal structure of B_2O_3, supporting the interpretation that boron preferentially forms stable B–O motifs rather than participating in direct metal–B bonding.

F. Characterization

Using a cryo-TEM holder working at -193 °C (tip temperature is -179 °C), we acquired aberration-corrected atomic-resolution annular dark-field scanning transmission electron microscopy (ADF-STEM) images of samples.

We have conducted synchrotron X-ray diffraction experiments as shown in Fig. S8. The resulting pair distribution functions (PDFs) and 2D diffraction patterns provide direct evidence of amorphization and further support the simulation results.

Compared with the crystalline LaB_6 standard, all three synthesized samples lack distinct Bragg reflections, confirming the absence of long-range order. Moreover, from Group-1 to Group-3, the broadening and fading of diffraction rings reflect increasing structural disorder with higher boron incorporation. The corresponding 1D intensity profiles and PDFs demonstrate the suppression of medium- and long-range correlations, consistent with amorphous behavior. These observations align with our simulations and further substantiate the claim that increasing boron content promotes amorphization.

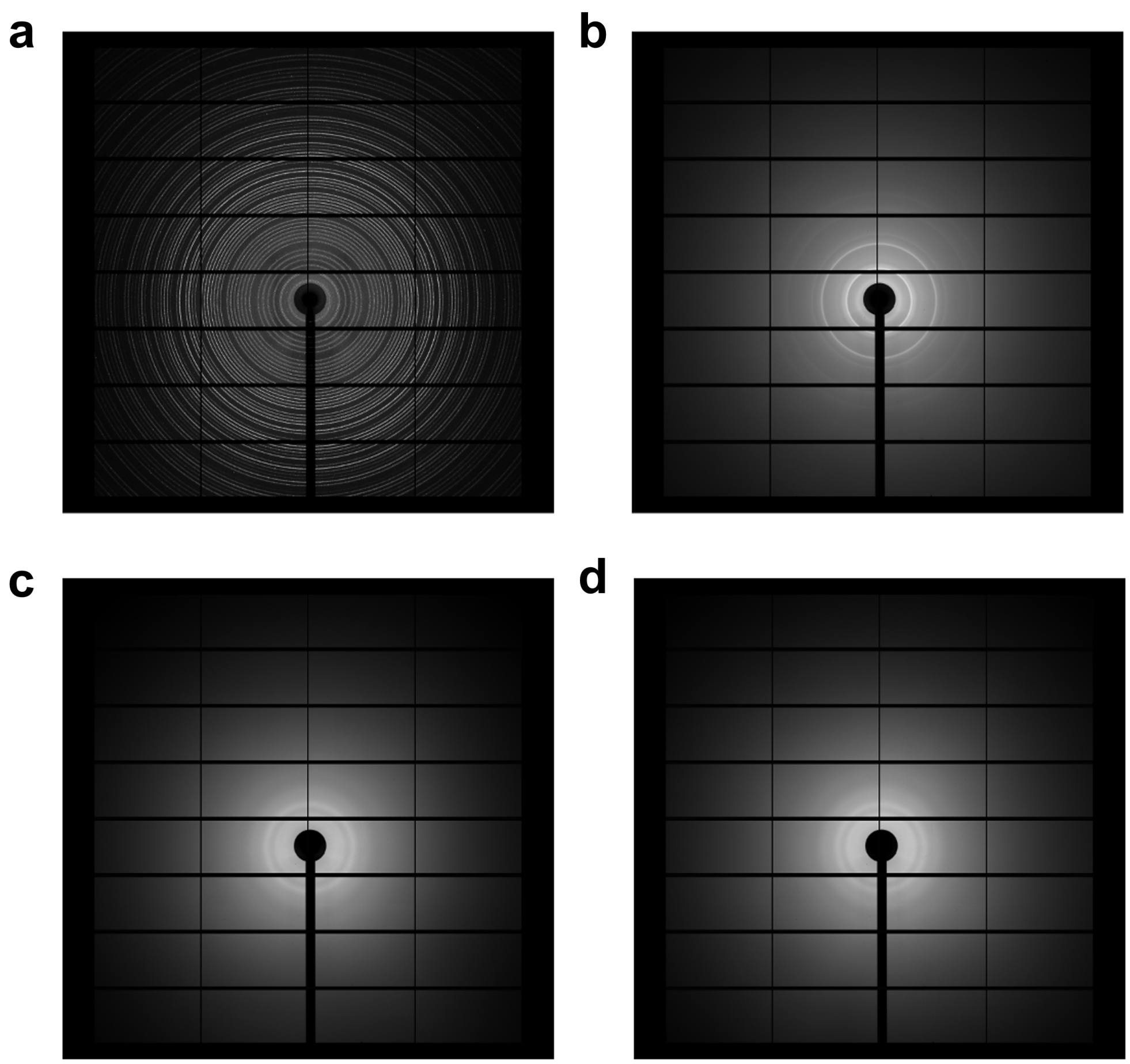

FIG. S8. **Synchrotron X-ray diffraction patterns of reference and experimental samples.** (**a**) Reference LaB_6 exhibits sharp and well-defined diffraction rings, characteristic of a crystalline standard. (**b–d**) Diffraction patterns of Group-1, Group-2, and Group-3 samples, respectively, showing a progressive loss of ring sharpness and increased halo-like features with higher boron content, indicating enhanced amorphization.

TABLE S4. **Elemental compositions of catalysts estimated from ICP and element analysis.** Metallic contents were determined by ICP, and O contents were detected by difference subtraction of mass conservation.

Catalyst	Fe (mol %)	Co (mol %)	Ni (mol %)	Mo (mol %)	B (mol %)	O (mol %)
Group-1 (theor.)	8.6	7.8	8.6	7.0	6.3	61.7
Group-1 (exp.)	8.7	8.2	8.3	6.9	5.9	62.0
Group-2 (theor.)	9.4	8.6	8.6	6.3	8.6	58.6
Group-2 (exp.)	9.0	8.6	8.6	6.5	8.5	58.8
Group-3 (theor.)	10.2	10.2	10.2	7.0	11.7	50.8
Group-3 (exp.)	10.4	10.0	9.9	7.2	11.7	50.8

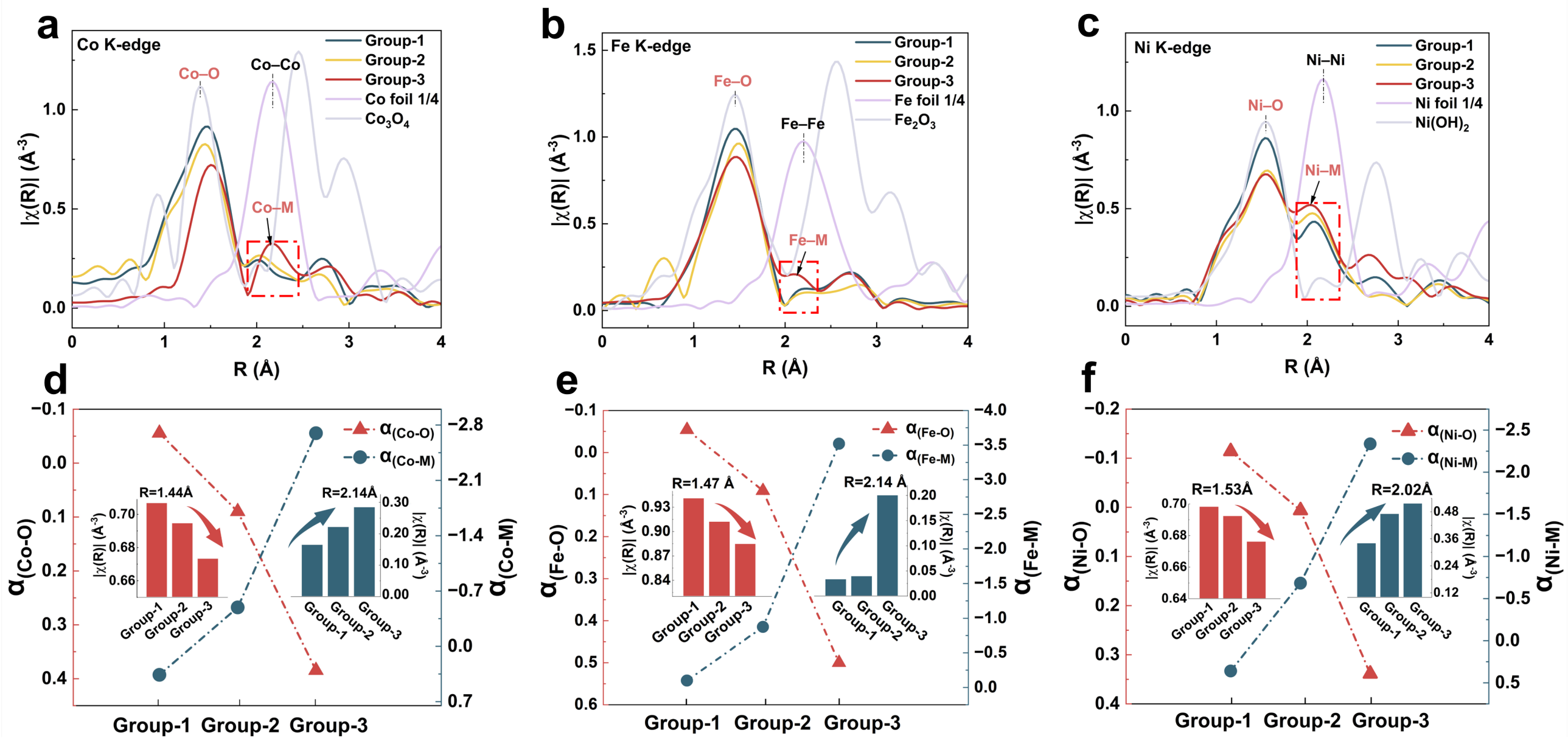

FIG. S9. **Structural characterization and interaction analysis.** (**a** to **c**) Fourier-transformed EXAFS spectra of Fe, Co, and Ni K-edges in R-space. (**d** to **f**) α_{ij} values for M–O and M–M (M = Co, Fe, Ni) interactions derived from predicted structures of $FeCoNiMoBO_x$ in three groups, compared with the magnitude of $|\chi|(R)$ from EXAFS. Positive α_{ij} values indicate dispersion, while negative values correspond to aggregation.

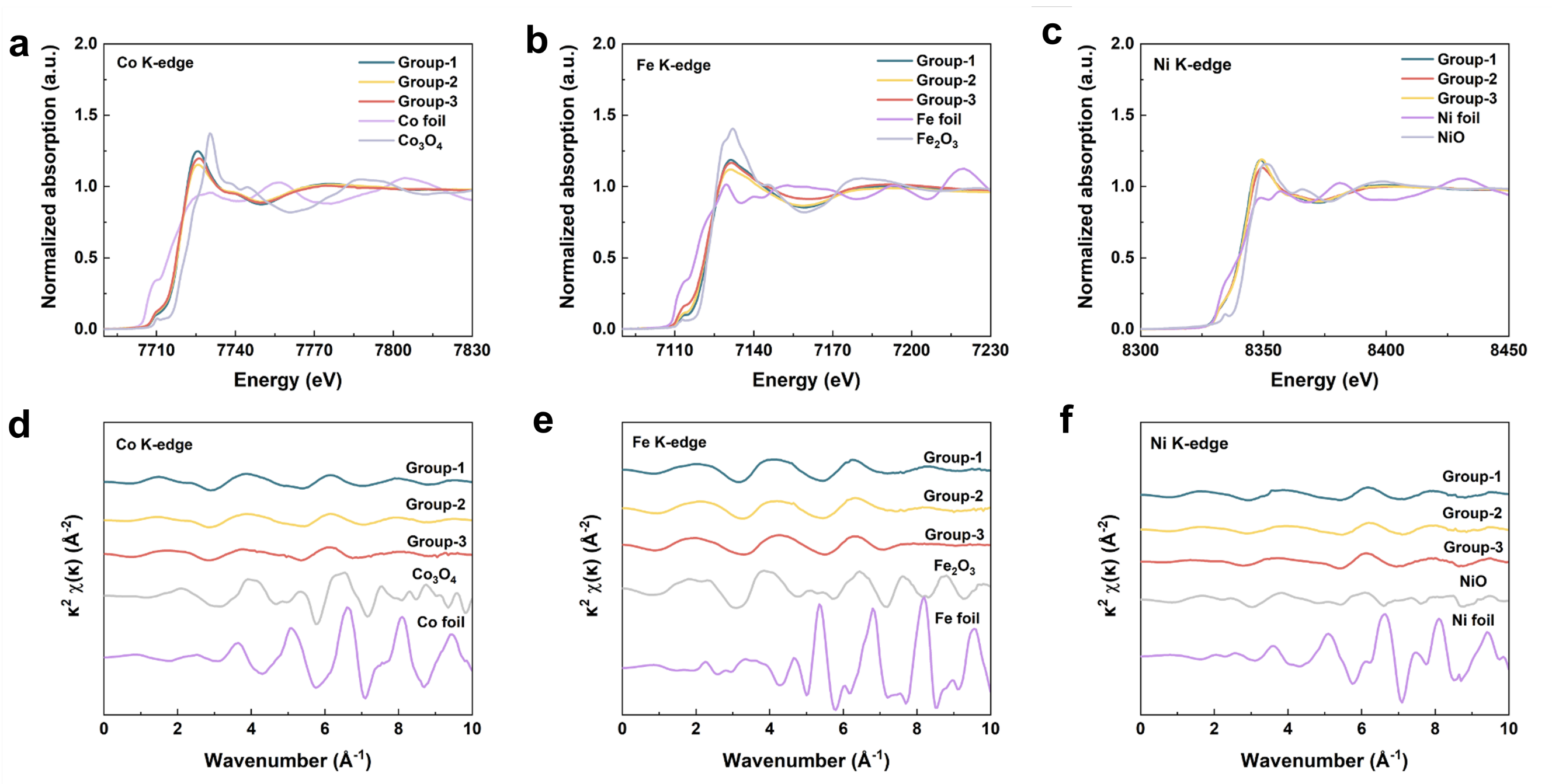

FIG. S10. **XAS and EXAFS analysis at the Co, Fe, and Ni K-edges.** (**a–c**) Normalized XANES spectra at the Co, Fe, and Ni K-edges for Group-1, Group-2, and Group-3, compared with corresponding metal foils and oxides. The absence of distinct post-edge features associated with Co–Co, Fe–Fe, and Ni–Ni coordination confirms the lack of long-range metal ordering. (**d–f**) Corresponding k-space EXAFS spectra ($k^2\chi(k)$). The dampened and broadened EXAFS signals in the experimental groups indicate increased structural disorder and amorphization relative to the crystalline standards.

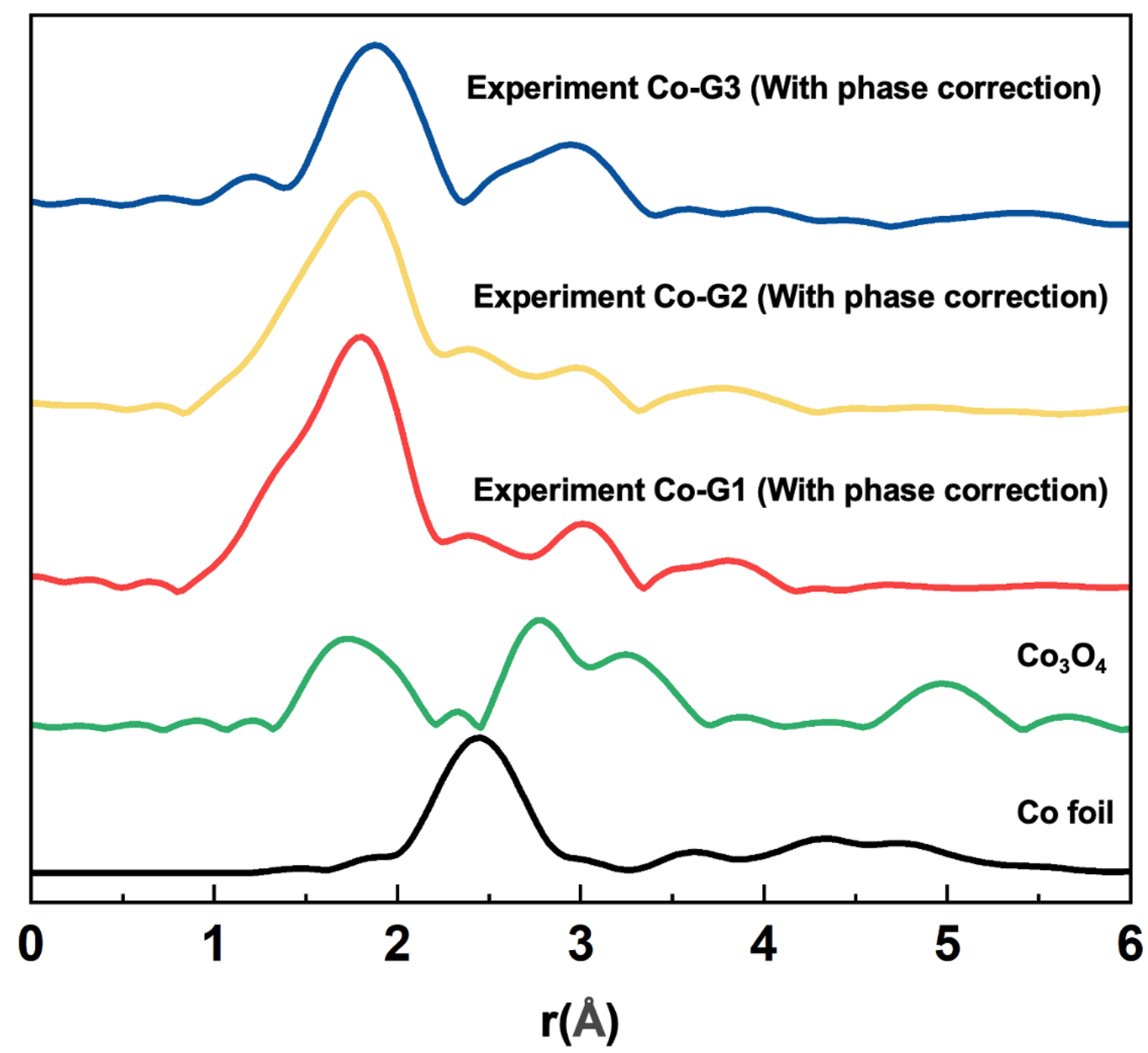

FIG. S11. **Fourier-transformed EXAFS spectra of Co-based samples.** Experimental Co-G1, Co-G2, and Co-G3 spectra after phase correction are compared with reference spectra of Co foil and Co_3O_4 in R-space. Phase correction aligns the experimental peaks with the actual interatomic distances, enabling accurate structural comparison of the local coordination environment around Co atoms.

TABLE S5. **Co K-edge EXAFS fitting parameters for FeCoNiMoBO$_x$ samples with varying boron contents.** Fitting results are shown for Group-1, Group-2, and Group-3, where M = Fe/Co/Ni/Mo. The coordination number (N), Debye–Waller factor (σ^2), and bond distance (R) are extracted from two-shell fits covering 1.00–2.40 Å (Co–O) and 2.50–3.85 Å (Co–M) ranges. With increasing boron content, $N_{\mathrm{Co-O}}$ decreases while $N_{\mathrm{Co-M}}$ increases, indicating a structural transition from oxygen-bridged Co–O–Co linkages toward direct metal–metal interactions. All σ^2 values remain physically reasonable, and low R-factors confirm good fitting quality.

Group	Name	N	σ^2	R (Å)	r-factor (%)
Group-1	O	1.000	0.01197	1.82359	0.01276
	O	2.914	0.01284	1.89610	
	O	2.383	0.00323	2.03618	
	M	0.478	0.00426	2.52069	
Group-2	O	2.000	0.01122	1.88906	0.02232
	O	1.500	0.00299	2.04353	
	O	1.110	0.00292	2.32660	
	M	0.621	0.00784	2.57409	
Group-3	O	1.641	0.00648	1.89911	0.31000
	O	1.354	0.00298	2.00070	
	O	0.600	0.02000	2.13367	
	M	1.000	0.00619	2.43548	

A controlled $FeCoNiMoBO_x$ series was synthesized using 5 M $NaBH_4$ solutions pre-hydrolyzed for 12, 24, 36, 48, and 72 h to examine the influence of the reducing-agent state on the chemical environment of boron. The metal-precursor concentrations, reagent-addition sequence, ice-bath temperature, stirring conditions, and reaction time were kept unchanged. As the pre-hydrolysis time increased, the low-binding-energy B 1*s* component near 188 eV progressively decreased and was no longer resolved after 72 h within the fitting uncertainty (Fig. S12a). This trend indicates that extended pre-hydrolysis suppresses the incorporation of reduced metal-boride-like species and favors oxidized B–O environments. Consistently, the final Group-1, Group-2, and Group-3 samples exhibit predominantly oxidized B–O components without a detectable M–B contribution (Fig. S12b). Their B *K*-edge XANES spectra are dominated by the BO_3-related feature; only a weak BO_4-related shoulder is observed, and no metal-boride-related feature is discernible.

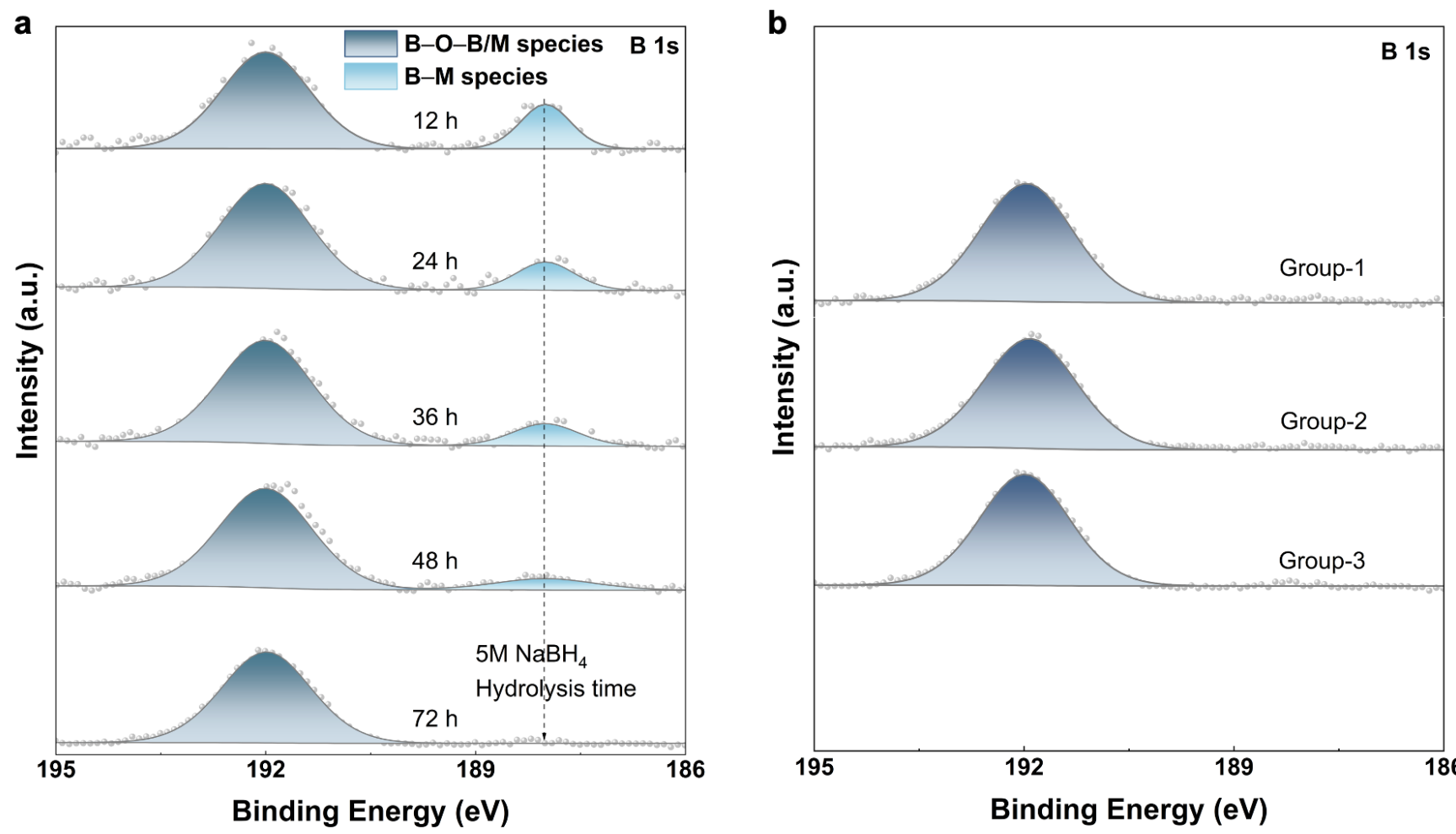

FIG. S12. **Influence of $NaBH_4$ pre-hydrolysis on the boron chemical state in $FeCoNiMoBO_x$.** (**a**) High-resolution B 1*s* XPS spectra of $FeCoNiMoBO_x$ samples synthesized using 5 M $NaBH_4$ solutions pre-hydrolyzed for 12, 24, 36, 48, and 72 h. The metal-boride-like contribution near 188 eV decreases with increasing pre-hydrolysis time and is not resolved after 72 h within the fitting uncertainty. (**b**) High-resolution B 1*s* XPS spectra of the final Group-1, Group-2, and Group-3 samples, showing predominantly oxidized B–O components.

G. Catalytic performance of Groups 1-3

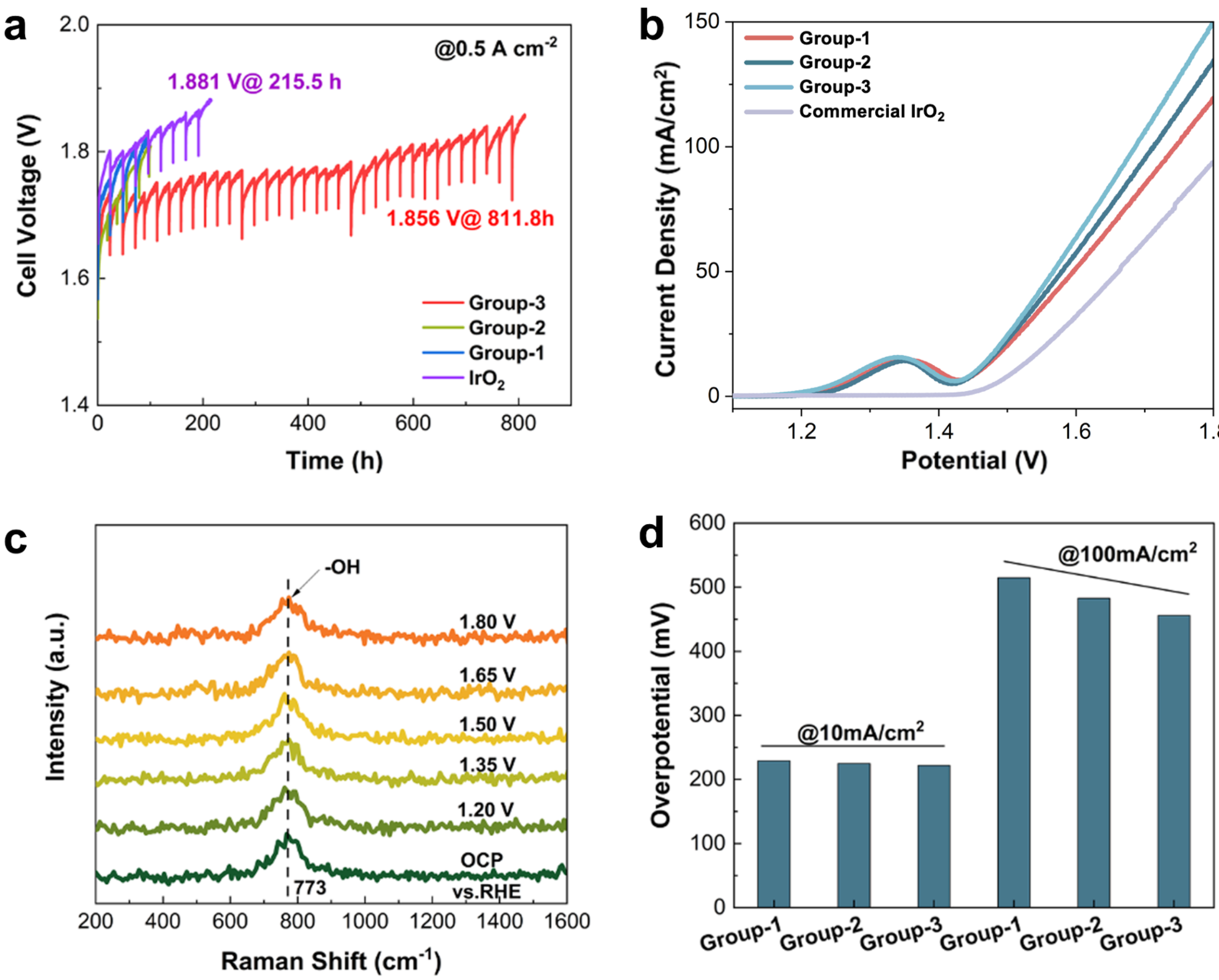

FIG. S13. **Catalytic stability.** (**a**) Stability test at 0.5 A/cm^2 using an anion exchange membrane (AEM) device for different groups, compared with IrO_2. (**b**) Linear sweep voltammetry curves of Group-1, -2, and -3, compared with commercial IrO2. (**c**) In-situ Raman spectra of FeCoNiMoBO$_X$ (Group-3). In situ Raman spectroscopy shows no detectable metal oxide vibrations (e.g., E_{2g}, A_{1g}) and a dominant −OH peak at 773 cm^{-1} persisting across applied potentials, indicating structural stability. (**d**) OER overpotentials across different groups at varying current densities.

Figure S13d highlights the distinction between low-current catalytic activity and high-current electrode performance. For the Group-3 catalyst, the uncompensated overpotential increases from 222 mV at 10 mA cm^{-2} to 456 mV at 100 mA cm^{-2}. The series resistance (R_s), determined from the high-frequency intercept of the EIS Nyquist plot, was 1.6 ohms. After applying an 80% iR correction, the overpotential at 100 mA cm^{-2} decreases to 328 mV, indicating that approximately 128 mV of the apparent high-current overpotential originates from uncompensated ohmic loss. Moreover, the diameter of the EIS arc progressively decreases from Group-1 to Group-3 (Fig. S14), demonstrating that Group-3 does not exhibit the highest interfacial charge-transfer resistance. This trend is consistent with the enhanced metal–metal nearest-neighbor correlations revealed by the structural simulations, which may strengthen electronic coupling between adjacent metal centers and facilitate interfacial charge transfer.

Nevertheless, the remaining polarization at 100 mA cm^{-2} cannot be attributed solely to the local short-range-order motif. At high current density, the measured electrode response reflects a combination of intrinsic OER kinetics, accessible electrochemical surface area, catalyst-layer thickness and porosity, electrolyte and ionic transport, and gas-bubble accumulation. The SRO-constrained generative framework was designed to identify structurally viable amorphous compositions and favorable local atomic correlations; it was not trained to optimize these electrode- and device-level transport parameters. Consequently, improved local SRO does not necessarily translate into breakthrough performance at industrially relevant current densities. Quantitative separation of the remaining contributions would require additional measurements, including current-dependent impedance analysis, Tafel analysis, electrochemically active-surface-area normalization, and systematic evaluation of the catalyst-layer architecture. Accordingly, the

Group-3 catalyst is described here as competitive with recently reported non-noble-metal OER catalysts rather than as a record-performing catalyst.

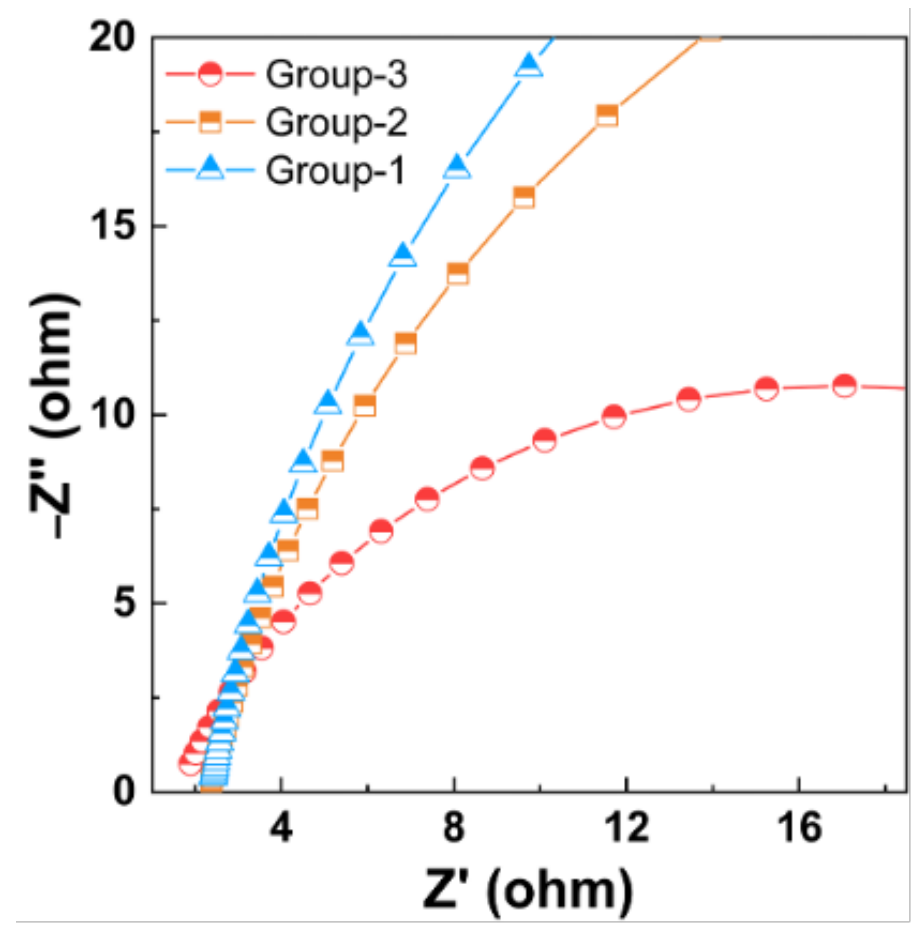

FIG. S14. **Electrochemical impedance spectroscopy of FeCoNiMoBO$_x$ catalysts.** Nyquist plots of the Group-1, Group-2, and Group-3 electrodes recorded at 1.53 V vs. RHE (η = 300 mV) in 1.0 M KOH. The progressively decreasing impedance-arc diameter from Group-1 to Group-3 indicates improved interfacial charge-transfer behavior.

To place the OER performance of FeCoNiMoBO$_x$ in the context of recently reported multimetal amorphous and high-entropy catalysts, Table S6 compares representative activity and durability metrics measured in 1.0 M KOH. The Group-3 FeCoNiMoBO$_x$ catalyst requires an overpotential of 222 mV at 10 mA cm^{-2} without iR compensation (209 mV with 80% iR correction). Complementarily, the catalyst sustains 500 mA cm^{-2} for 811 h in an AEM water electrolyzer, with a voltage increase rate of 0.227 mV h^{-1}, compared with 0.9 mV h^{-1} for IrO_2. This high-current test demonstrates favorable device-level durability but does not independently isolate the intrinsic catalytic kinetics. Because the literature values were obtained using different electrode preparations, catalyst loadings, cell configurations, and compensation protocols, the comparison should be interpreted as literature benchmarking rather than as a strictly protocol-normalized ranking.

TABLE S6. **Representative OER benchmarks for multimetal amorphous and high-entropy oxide/(oxy)hydroxide catalysts in 1.0 M KOH.** Auto denotes automatic iR correction by the electrochemical workstation.

**Catalyst**	**η_{10} (mV)**	**iR treatment**	**Stability**	**Reference**
Amorphous FeCoNiMoBO$_x$	222 (209)	Uncompensated (80% iR-corrected)	811 h @ 500 mA cm^{-2} (0.227 mV h^{-1} voltage increase-rate)	This work
Amorphous Ru@NiMoO$_x$	220	95% iR-corrected	100 h @ 1000 mA cm^{-2}	*Nat. Commun.* **16**, 8827 (2025)
Amorphous NiB	343	Auto iR-corrected	115 h, > 80 mA cm^{-2} @ 1.73 V vs. RHE	*J. Mater. Chem. A* **13**, 7962–7972 (2025)
Partially amorphous Co–B oxides	338	85% iR-corrected	48 h @ 10 mA cm^{-2}	*Angew. Chem. Int. Ed.* **61**, 42 (2022)
Ni-POM@CoFe-MOF	178	Auto iR-corrected	1000 h, ∼100 mA cm^{-2} (chronoamperometry)	*Science* **388**, 430–436 (2025)
High-entropy oxyhydroxide MoZnFeCoNiOOH	221	Auto iR-corrected	1600 h @ 100 mA cm^{-2}	*Sci. Adv.* **10**, eadq6758 (2024)
High-entropy oxyhydroxide Ag@CoCuFeAgMoOOH	218	Auto iR-corrected	50 h @ 10 mA cm^{-2}	*Adv. Mater.* **34**, 2110511 (2022)
High-entropy LDH Au@MnFeCoNiCu	213	95% iR-corrected	700 h, ∼100 mA cm^{-2} @ 1.53 V vs. RHE	*Nat. Commun.* **14**, 6019 (2023)

H. Catalytic descriptor calculation

To quantify the electronic factors governing the oxygen evolution reaction (OER) activity, we adopted two Co-centered descriptors: the unoccupied d-band state parameter (λ_1) and the active d-band state parameter (λ_2). These two parameters characterize, respectively, the density of unoccupied Co d states at the active site and the number of electronically active states available for interfacial charge transfer. Because operando spectroscopy indicates that Co undergoes pronounced oxidation under OER conditions, Co-centered electronic descriptors provide an effective way to correlate the local electronic structure with the catalytic performance of $FeCoNiMoBO_x$ catalysts with different boron contents.

Specifically, λ_1 and λ_2 were obtained from the projected density of states (PDOS) by integrating the relevant electronic states as follows:

$$\begin{aligned} \lambda_1 &= N_{\mathrm{Co}\,3d_{\mathrm{unoccupied}}} + \frac{N_{\mathrm{Co}\,(d_{x^2-y^2},\,d_{z^2})_{\mathrm{unoccupied}}}}{N_{\mathrm{O}\,2p_{\mathrm{unoccupied}}}}, \\ \lambda_2 &= N_{\mathrm{Co}\,3d} \times \frac{N_{\mathrm{Co}\,(d_{x^2-y^2},\,d_{z^2})_{\mathrm{unoccupied}}}}{N_{\mathrm{O}\,2p_{\mathrm{unoccupied}}}}. \end{aligned} \tag{S2}$$

Here, $N_{\mathrm{Co}\,3d}$ denotes the total number of states in the Co $3d$ band, $N_{\mathrm{Co}\,3d_{\mathrm{unoccupied}}}$ is the number of unoccupied states in the Co $3d$ band, $N_{\mathrm{Co}\,(d_{x^2-y^2},\,d_{z^2})_{\mathrm{unoccupied}}}$ represents the number of unoccupied states in the Co $d_{x^2-y^2}$ and d_{z^2} orbitals, and $N_{\mathrm{O}\,2p_{\mathrm{unoccupied}}}$ is the number of unoccupied states in the O $2p$ band. Physically, λ_1 reflects the extent of unoccupied Co d states that can accept electrons during the adsorption and transformation of oxygenated intermediates. A larger λ_1 therefore indicates a more favorable electronic configuration for charge redistribution at the catalytic site. In contrast, λ_2 describes the number of Co d states that can actively participate in electron-transfer interactions with O $2p$ orbitals under relatively weak metal–oxygen coupling. Higher λ_2 values correspond to a greater number of electronically active states and are likewise expected to favor OER kinetics and reduce the overpotential.

In situ near-ambient-pressure X-ray photoelectron spectroscopy (NAP-XPS) measurements using synchrotron radiation were performed to clarify the main active sites in the high-entropy catalysts. In the field of oxygen-evolution catalysis, there is broad consensus that multi-metal oxides containing Fe, Co, and Ni outperform their single-metal counterparts (57, 58). Among the possible active motifs, CoOOH domains have been identified as especially effective in lowering the activation barrier of the rate-determining step (24). The *in situ* NAP-XPS measurements (Fig. S15a) on the most active Group-3 $FeCoNiMoBO_x$ catalyst show that the Co $2p$ spectra (Fig. S15b) undergo pronounced changes under OER-relevant potentials, including splitting of the $2p_{3/2}$ peak and the appearance of a high-binding-energy shoulder characteristic of CoOOH. In contrast, the Fe $2p$ and Ni $2p$ spectra (Figs. S15c and S15d) remain essentially unchanged, indicating that Co sites act as the dominant adsorption centers for OER intermediates, especially at potentials close to thermodynamic equilibrium. Accordingly, descriptors derived from the Co projected density of states (PDOS) are expected to be particularly relevant for capturing the composition-dependent activity trend.

The calculated descriptor trends are summarized in Fig. S16. As the boron content increases from 6% to 12%, both λ_1 and λ_2 increase monotonically, whereas the experimentally measured overpotential decreases. This inverse correlation indicates that the OER performance is closely linked to the availability of Co-centered unoccupied and transfer-active d states. In particular, a larger λ_1 corresponds to a higher density of accessible unoccupied Co d states that can accommodate electron redistribution during the formation and conversion of oxygen-containing intermediates. Similarly, a larger λ_2 indicates a greater number of active Co d states that can participate in orbital interaction and charge transfer with O $2p$ states.

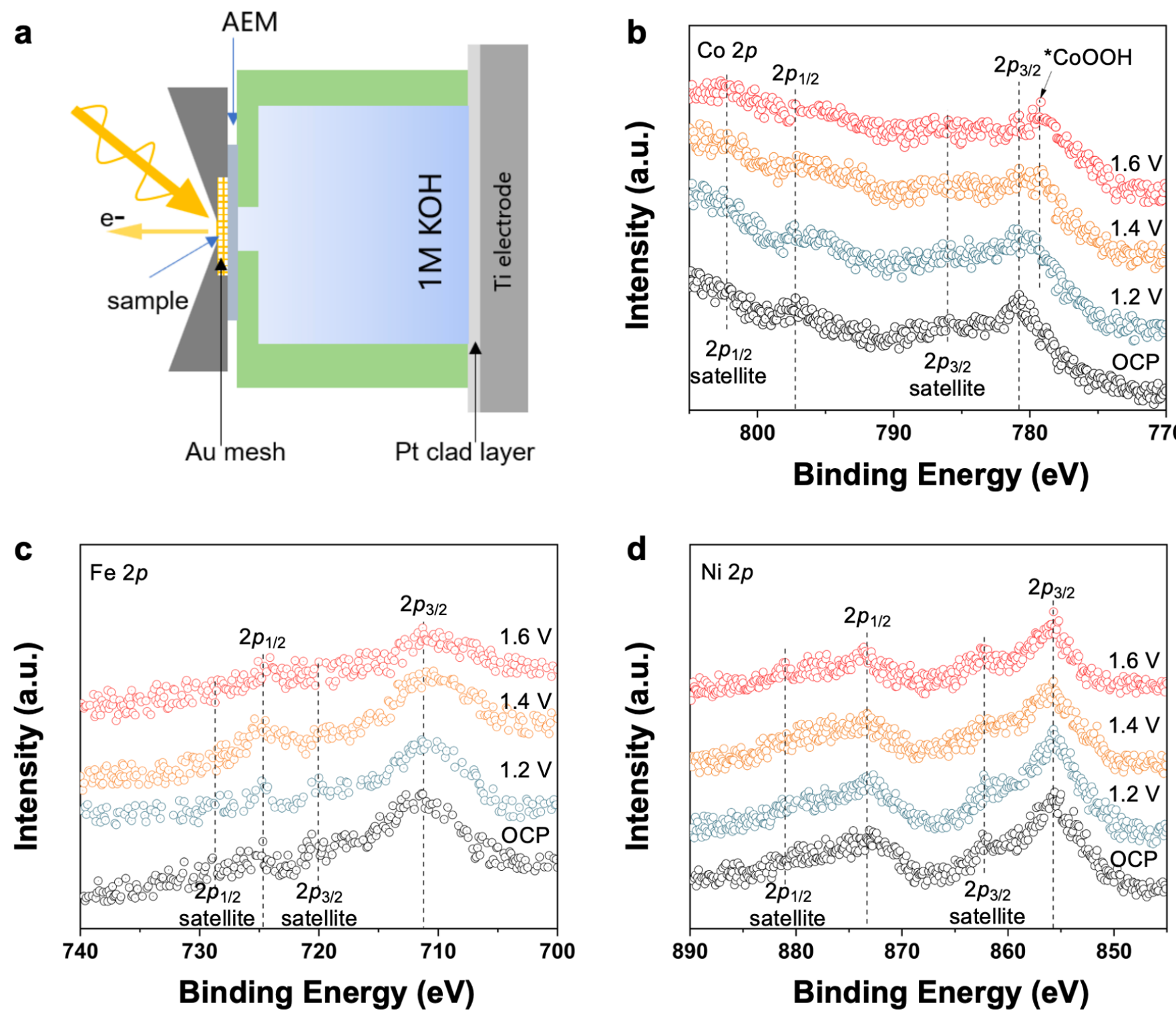

FIG. S15. **In situ near-ambient-pressure X-ray photoelectron spectroscopy (NAP-XPS) using synchrotron radiation sources.** (a) Schematic diagram of the in-situ reaction cell. (b) Evolution of Co 2p photoemission spectra, (c) Fe 2p spectra, and (d) Ni 2p spectra of the Group-3 sample recorded at different applied potentials (OCP, 1.2 V, 1.4 V, and 1.6 V versus RHE) in 1 M KOH. Characteristic 2$p_{1/2}$ and 2$p_{3/2}$ peaks, along with satellite features, are labeled.

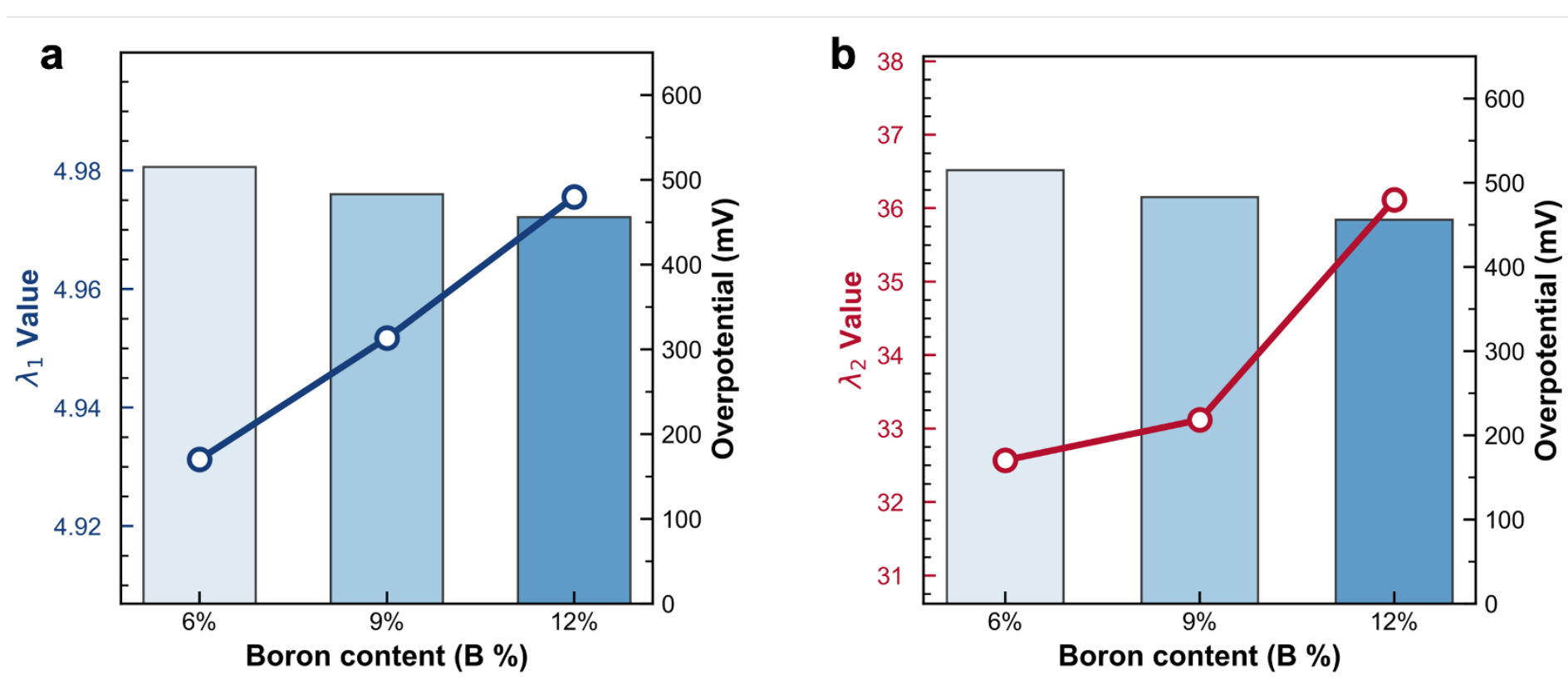

FIG. S16. **Correlation between Co-centered electronic descriptors and OER activity as a function of boron content.** (a) Calculated unoccupied d-band state parameter (λ_1) of Co sites and the corresponding overpotential for FeCoNiMoBO$_x$ catalysts with different boron contents. (b) Calculated active d-band state parameter (λ_2) of Co sites and the corresponding overpotential as a function of boron content.

I. Detailed synthesis and electrochemical methods

We developed a concise synthetic route for intrinsically highly amorphous MBO_x catalysts. The strategy is not limited to a single $FeCoNiMoBO_x$ composition, but extends across a broad multicomponent sample space that includes binary, ternary, quaternary, quinary, octary, and decenary systems, and potentially compositions of even greater complexity. The small covalent radius and electron-deficient character of boron enable it to disrupt crystalline periodicity and promote the formation of stable, coordination-flexible amorphous matrices capable of hosting multiple transition-metal centers.

To realize this concept, the quinary $FeCoNiMoBO_x$ system was used as a representative example. Equimolar amounts of $Fe(NO_3)_3$, $Co(NO_3)_2$, $Ni(NO_3)_2$, and $(NH_4)_6Mo_7O_{24}$ were dissolved to prepare 0.2 M precursor solutions, which were stored at 4 °C. A separately prepared 5 M $NaBH_4$ solution was likewise refrigerated for 72 h to allow partial spontaneous hydrolysis. In an ice bath, the metal-salt solutions were first mixed, followed by rapid addition of hydrolyzed $NaBH_4$ at half the total metal-solution volume under vigorous stirring. After reaction for 3, 6, or 18 h, three distinct $FeCoNiMoBO_x$ catalysts, denoted as Group-1, Group-2, and Group-3, respectively, were isolated.

The other catalysts were synthesized using the same molar ratios listed in the table below and following the same synthetic procedure, except that the reaction was allowed to proceed for 18 h to ensure completion. The use of highly concentrated hydrolyzed $NaBH_4$ is considered critical because its hydrolysis creates a strongly alkaline environment enriched with boron–oxygen species and reducing borohydride-derived complexes. Upon introduction of the metal ions, the abundant boron–oxygen species suppress rapid metal migration while simultaneously coupling with the metal centers. Meanwhile, boron species derived from the reducing agent can also be incorporated into the metal-oxide matrix. These synergistic effects collectively promote the formation of amorphous metal borate/oxide products.

TABLE S7. Precursor volumes used for synthesizing amorphous multi-metal catalysts of different compositional complexities.

Catalysts	Metal salt-A (0.2 M)	M-B	M-C	M-D	M-E	M-F	M-G	M-H	M-I	M-J	Hydrolyzed $NaBH_4$ (5 M)
Binary	5 mL	5 mL	–	–	–	–	–	–	–	–	5 mL
Ternary	5 mL	5 mL	5 mL	–	–	–	–	–	–	–	7.5 mL
Quaternary	5 mL	5 mL	5 mL	5 mL	–	–	–	–	–	–	10 mL
Quinary	5 mL	5 mL	5 mL	5 mL	5 mL	–	–	–	–	–	12.5 mL
Octary	5 mL	5 mL	5 mL	5 mL	5 mL	5 mL	5 mL	5 mL	–	–	20 mL
Denary	5 mL	5 mL	5 mL	5 mL	5 mL	5 mL	5 mL	5 mL	5 mL	5 mL	25 mL

TABLE S8. **Synthesized compositions and corresponding characterization.**

**Composition**	**Structural characterization**	**Electrochemical evaluation**	**Purpose in study**
$FeMoBO_x$	XRD	LSV	Binary-metal platform
$CoMoBO_x$	XRD	LSV	Binary-metal platform
$NiMoBO_x$	XRD	LSV	Binary-metal platform
$FeCoMoBO_x$	XRD	LSV	Ternary-metal platform
$FeNiMoBO_x$	XRD	LSV	Ternary-metal platform
$CoNiMoBO_x$	XRD	LSV	Ternary-metal platform
$FeCoNiBO_x$	XRD	LSV	Ternary-metal platform
$FeCoNiMoBO_x$	XRD; synchrotron XRD/PDF; B K-edge XANES; metal K-edge XANES/EXAFS; ICP-OES; operando spectroscopy	LSV; high-current polarization; AEMWE durability	Representative system for detailed theory–experiment validation
MnFeCoNiCuZnMo			
$SnBO_x$	XRD	LSV	Eight-metal extension
AlMnFeCoNiCuZnMo			
$SnWBO_x$	XRD; ADF-STEM/EDS mapping	LSV	Ten-metal extension and homogeneity demonstration

Three-electrode electrochemical measurements. All electrochemical tests were performed using a CHI760E electrochemical workstation with a three-electrode configuration. The working electrode consisted of carbon paper (CP) loaded with the catalyst. To prepare the working electrode, the catalyst was first dispersed in a mixed solvent of deionized water and isopropanol (1:1 v/v) to form an ink with a concentration of 5 $mg\,mL^{-1}$. The ink was then deposited onto the carbon paper to achieve a catalyst loading of 0.5 $mg\,cm^{-2}$ over a geometrically exposed area of 1.0 cm^2. A Hg/HgO electrode and a Pt foil served as the reference and counter electrodes, respectively, and the electrolyte was 1.0 M KOH. Linear sweep voltammetry (LSV) polarization curves were recorded at 25 °C with a scan rate of 5 $mV\,s^{-1}$. Electrochemical impedance spectroscopy (EIS) was performed at an applied potential of 1.53 V vs. RHE over a frequency range from 100 kHz to 0.1 Hz. The series resistance (R_s) was determined from the high-frequency intercept of the EIS Nyquist plot and was 1.6 ohms for the Group-3 electrode. Both uncompensated and post-measurement iR-corrected polarization data are reported. An 80% iR correction was applied according to

$$E_{corr} = E_{meas} - 0.80\, iR_s, \tag{S3}$$

where i is the total electrode current rather than the current density. At 100 $mA\,cm^{-2}$, the total current through the 1.0 cm^2 electrode was 0.100 A, corresponding to a correction of 128 mV. The uncompensated and corrected values are reported separately to distinguish the as-measured electrode response from the contribution of uncompensated series resistance.

Anion-exchange membrane water electrolyzer (AEMWE) stability testing. For AEM-based electrolyzer tests, the anode was sprayed with the $FeCoNiMoBO_x$ catalyst at a loading of 3 $mg\,cm^{-2}$, while the cathode was coated with 20 wt% Pt/C at a loading of 0.05 $mg\,cm^{-2}$. The electrolyte was 1.0 M KOH, and the measurements were conducted at 60 °C.

REFERENCES

1. Y. Liu, A. Madanchi, A. S. Anker, L. Simine, V. L. Deringer, The amorphous state as a frontier in computational materials design. *Nat. Rev. Mater.* **10**, 228–241 (2024).

2. B. Wu, X. Liu, P. Liu, G. Wu, L. Tian, X. Han, J. Li, X. Hong, Synthesis of amorphous metal oxides via a crystalline to amorphous phase transition strategy. *Nat. Synth.* **4**, 370–379 (2025).

3. C. A. Schuh, T. C. Hufnagel, U. Ramamurty, Mechanical behavior of amorphous alloys. *Acta Mater.* **55**, 4067–4109 (2007).

4. M. Sakata, N. Cowlam, H. A. Davies, Chemical short-range order in liquid and amorphous $Cu_{66}Ti_{34}$ alloys. *J. Phys. F* **11**, L157–L162 (1981).

5. X. Chen, Q. Wang, Z. Cheng, M. Zhu, H. Zhou, P. Jiang, L. Zhou, Q. Xue, F. Yuan, J. Zhu, X. Wu, E. Ma, Direct observation of chemical short-range order in a medium-entropy alloy. *Nature* **592**, 712–716 (2021).

6. C. K. Saw, R. B. Schwarz, Chemical short-range order in dense random-packed models. *J. Less Common Met.* **140**, 385–393 (1988).

7. H. W. Sheng, W. K. Luo, F. M. Alamgir, J. M. Bai, E. Ma, Atomic packing and short-to-medium-range order in metallic glasses. *Nature* **439**, 419–425 (2006).

8. H. Yang, B. Yan, Y. Xue, T. Guo, Z. Wang, G. Teobaldi, P. Hu, L.-M. Liu, L. Guo, Amorphous monolayer CuPd catalysts for selective semihydrogenation. *Sci. Adv.* **11**, eadx8081 (2025).

9. Y. Zhao, S. Gao, X. Chen, Y. Liu, M. Zhang, Y. Liu, J. Kang, T. Guo, L.-M. Liu, L. Guo, In situ identification of dynamic active sites in the electrocatalytic nitrate reduction reaction. *J. Am. Chem. Soc.* **147**, 31722–31730 (2025).

10. R. Li, S. Yang, Y. Zhang, G. Yu, C. Wang, C. Chen, G. Wu, R. Sun, G. Wang, X. Zheng, W. Yan, G. Wang, D. Rao, X. Hong, Short-range order in amorphous nickel oxide nanosheets enables selective and efficient electrochemical hydrogen peroxide production. *Cell Rep. Phys. Sci.* **3**, 100788 (2022).

11. K. Zhang, T. Yang, T. Chen, Y. Yang, Z. Jiang, C. Gao, Y. Zuo, W. Xiao, D. Xia, An amorphous Li–V–O–F cathode with tetrahedral coordination and O–O formal redox at low voltage. *Nat. Mater.* **24**, 1600–1607 (2025).

12. F. H. Stillinger, A topographic view of supercooled liquids and glass formation. *Science* **267**, 1935–1939 (1995).

13. E. Holmström, B. Haberl, O. Pakarinen, K. Nordlund, F. Djurabekova, R. Arenal, J. Williams, J. Bradby, T. Petersen, A. Liu, Dependence of short and intermediate-range order on preparation in experimental and modeled pure a-Si. *J. Non Cryst. Solids* **438**, 26–36 (2016).

14. J. K. Christie, Review: Understanding the properties of amorphous materials with high-performance computing methods. *Philos. Trans. R. Soc. London Ser. A Math. Phys. Eng. Sci.* **381**, 20220251 (2023).

15. S. Amokrane, A. Ayadim, L. Levrel, Structure of the glass-forming metallic liquids by ab initio and classical molecular dynamics, a case study: Quenching the $Cu_{60}Ti_{20}Zr_{20}$ alloy. *J. Appl. Phys.* **118**, 194903 (2015).

16. V. L. Deringer, N. Bernstein, A. P. Bartók, M. J. Cliffe, R. N. Kerber, L. E. Marbella, C. P. Grey, S. R. Elliott, G. Csányi, Realistic atomistic structure of amorphous silicon from machine-learning-driven molecular dynamics. *J. Phys. Chem. Lett.* **9**, 2879–2885 (2018).

17. Z. Zhang, J. Ding, E. Ma, Shear transformations in metallic glasses without excessive and predefinable defects. *Proc. Natl. Acad. Sci. U.S.A.* **119**, e2213941119 (2022).

18. K. Vollmayr, W. Kob, K. Binder, How do the properties of a glass depend on the cooling rate? A computer simulation study of a Lennard-Jones system. *J. Chem. Phys.* **105**, 4714–4728 (1996).

19. Y. Zhang, F. Zhang, C. Z. Wang, M. I. Mendelev, M. J. Kramer, K. M. Ho, Cooling rates dependence of medium-range order development in $Cu_{64.5}Zr_{35.5}$ metallic glass. *Phys. Rev. B* **91**, 064105 (2015).

20. J. Ashwin, E. Bouchbinder, I. Procaccia, Cooling-rate dependence of the shear modulus of amorphous solids. *Phys. Rev. E Stat. Nonlin. Soft Matter Phys.* **87**, 042310 (2013).

21. R. Su, S. Zhang, X. Zhang, Y. Yang, W. Wang, P. Guan, Atomic origin of annealing embrittlement in metallic glasses. arXiv:2208.13747 [cond-mat.mtrl-sci] (2022).

22. H. I. Schlesinger, H. C. Brown, A. E. Finholt, J. R. Gilbreath, H. R. Hoekstra, E. K. Hyde, Sodium borohydride, its hydrolysis and its use as a reducing agent and in the generation of hydrogen. *J. Am. Chem. Soc.* **75**, 215–219 (1953).

23. C. Liu, T. Yang, S. Li, Y. Wu, Q. Jiang, J. Xie, Y. Xu, Y. Bu, C. Peng, L. Tang, A. Mahmood, J. Zhou, B. Xu, H. Xiao, M. Li, Amorphization induces high-density undercoordinated indium sites for enhanced electrocatalytic urea synthesis. *ACS Catal.* **15**, 8489–8496 (2025).

24. B. Zhang, X. Zheng, O. Voznyy, R. Comin, M. Bajdich, M. García-Melchor, L. Han, J. Xu, M. Liu, L. Zheng, F. P. G. de Arquer, C. T. Dinh, F. Fan, M. Yuan, E. Yassitepe, N. Chen, T. Regier, P. Liu, Y. Li, P. D. Luna, A. Janmohamed, H. L. Xin, H. Yang, A. Vojvodic, E. H. Sargent, Homogeneously dispersed multimetal oxygen-evolving catalysts. *Science* **352**, 333–337 (2016).

25. G. Chen, W. Zhou, D. Guan, J. Sunarso, Y. Zhu, X. Hu, W. Zhang, Z. Shao, Two orders of magnitude enhancement in oxygen evolution reactivity on amorphous $Ba_{0.5}Sr_{0.5}Co_{0.8}Fe_{0.2}O_{3-\delta}$ nanofilms with tunable oxidation state. *Sci. Adv.* **3**, e1603206 (2017).

26. B. Zhang, L. Wang, Z. Cao, S. M. Kozlov, F. P. García de Arquer, C. T. Dinh, J. Li, Z. Wang, X. Zheng, L. Zhang, Y. Wen, O. Voznyy, R. Comin, P. De Luna, T. Regier, W. Bi, E. E. Alp, C.-W. Pao, L. Zheng, Y. Hu, Y. Ji, Y. Li, Y. Zhang, L. Cavallo, H. Peng, E. H. Sargent, High-valence metals improve oxygen evolution reaction performance by modulating 3d metal oxidation cycle energetics. *Nat. Catal.* **3**, 985–992 (2020).

27. G. Ferlat, A. P. Seitsonen, M. Lazzeri, F. Mauri, Hidden polymorphs drive vitrification in B_2O_3. *Nat. Mater.* **11**, 925–929 (2012).

28. W.-L. Li, X. Chen, T. Jian, T.-T. Chen, J. Li, L.-S. Wang, From planar boron clusters to borophenes and metalloborophenes. *Nat. Rev. Chem.* **1**, 0071 (2017).

29. D. Zhang, X. Liu, X. Zhang, C. Zhang, C. Cai, H. Bi, Y. Du, X. Qin, A. Peng, J. Huang, B. Li, Y. Shan, J. Zeng, Y. Zhang, S. Liu, Y. Li, J. Chang, X. Wang, S. Zhou, J. Liu, X. Luo, Z. Wang, W. Jiang, J. Wu, Y. Yang, J. Yang, M. Yang, F.-Q. Gong, L. Zhang, M. Shi, F.-Z. Dai, D. M. York, S. Liu, T. Zhu, Z. Zhong, J. Lv, J. Cheng, W. Jia, M. Chen, G. Ke, E. Weinan, L. Zhang, H. Wang, DPA-2: A large atomic model as a multi-task learner. *NPJ Comput. Mater.* **10**, 293 (2024).

30. G. C. Sosso, J. Chen, S. J. Cox, M. Fitzner, P. Pedevilla, A. Zen, A. Michaelides, Crystal nucleation in liquids: Open questions and future challenges in molecular dynamics simulations. *Chem. Rev.* **116**, 7078–7116 (2016).

31. S. An, Y. Li, J. Li, S. Zhao, B. Liu, P. Guan, The linear relationship between diffusivity and crystallization kinetics in a deeply supercooled liquid $Ni_{50}Ti_{50}$ alloy. *Acta Mater.* **152**, 1–6 (2018).

32. W. Zhou, J. Song, L. Lin, H. Yang, S. Guo, G. Ran, Y. Wang, Amorphization evolution study of CrCoFeNiMn high entropy alloy for mechanical performance optimization by deep potential molecular dynamics. *NPJ Comput. Mater.* **11**, 69 (2025).

33. S. Bakhtiari, A. Agrawal, H. Yang, R. Mirzaeifar, L. Cui, D. Jiang, Y. Liu, Third element diffusion induced amorphization of NiTi in a NiTi-nb nanocomposite. *Acta Mater.* **257**, 119180 (2023).

34. C. C. Yuan, F. Yang, F. Kargl, D. Holland-Moritz, M. Z. Li, X. L. Wang, B. Zhang, W. H. Wang, A. Meyer, Sluggish dynamics in Al-containing metallic glass-forming melts. *Acta Mater.* **285**, 120652 (2025).

35. L. P. Kreuzer, F. Yang, A. Meyer, N. Jakse, Impact of local structure on melt dynamics in Cu–Ti alloys: Insights from abinitio molecular dynamics simulations. *Phys. Rev. B* **111**, 144107 (2025).

36. K. Yang, H. Qin, H. Huang, Y. Zhu, Y. Lü, Microscopic dynamics of enhanced glass-forming ability with minor oxygen addition in bulk metallic glasses. *J. Chem. Phys.* **162**, 054503 (2025).

37. E. P. George, D. Raabe, R. O. Ritchie, High-entropy alloys. *Nat. Rev. Mater.* **4**, 515–534 (2019).

38. K.-Y. Tsai, M.-H. Tsai, J.-W. Yeh, Sluggish diffusion in Co–Cr–Fe–Mn–Ni high-entropy alloys. *Acta Mater.* **61**, 4887–4897 (2013).

39. P. Kuhn, J. Horbach, F. Kargl, A. Meyer, T. Voigtmann, Diffusion and interdiffusion in binary metallic melts. *Phys. Rev. B* **90**, 024309 (2014).

40. C. G. Schön, M. A. Tunes, R. Arroyave, J. Agren, On the complexity of solid-state diffusion in highly concentrated alloys and the sluggish diffusion core-effect. *Calphad* **68**, 101713 (2020).

41. R. Reali, J. A. Van Orman, J. S. Pigott, J. M. Jackson, F. Boioli, P. Carrez, P. Cordier, The role of diffusion-driven pure climb creep on the rheology of bridgmanite under lower mantle conditions. *Sci. Rep.* **9**, 2053 (2019).

42. M. E. Fleet, S. Muthupari, Coordination of boron in alkali borosilicate glasses using XANES. *J. Non Cryst. Solids* **255**, 233–241 (1999).

43. M. E. Fleet, S. Muthupari, Boron K-edge XANES of borate and borosilicate minerals. *Am. Mineral.* **85**, 1009–1021 (2000).

44. M. E. Fleet, X. Liu, Boron K-edge XANES of boron oxides: Tetrahedral B-O distances and near-surface alteration. *Phys. Chem. Miner.* **28**, 421–427 (2001).

45. J. M. Cowley, An approximate theory of order in alloys. *Phys. Rev.* **77**, 669–675 (1950).

46. F. Fan, B. Lei, X. Song, J. Liang, W. Cai, Y. Zhong, Y. Wu, N. Bao, L. Zhang, Applicable descriptors under weak metal-oxygen d-p interaction for the oxygen evolution reaction. *Angew. Chem. Int. Ed. Engl.* **64**, e202419718 (2025).

47. V. A. Mints, J. K. Pedersen, J. C. Olsen, M. K. Plenge, M. Arenz, J. Rossmeisl, Unraveling the mixing entropy-activity relationship in high entropy alloy catalysts: The more, the better?. *J. Am. Chem. Soc.* **148**, 4815–4825 (2026).

48. D. de Fontaine, The number of independent pair-correlation functions in multicomponent systems. *J. Appl. Cryst.* **4**, 15–19 (1971).

49. A. V. Ceguerra, M. P. Moody, R. C. Powles, T. C. Petersen, R. K. W. Marceau, S. P. Ringer, Short-range order in multicomponent materials. *Acta Crystallogr. A* **68**, 547–560 (2012).

50. J. Behler, M. Parrinello, Generalized neural-network representation of high-dimensional potential-energy surfaces. *Phys. Rev. Lett.* **98**, 146401 (2007).

51. X. Luo, Z. Wang, P. Gao, J. Lv, Y. Wang, C. Chen, Y. Ma, Deep learning generative model for crystal structure prediction. *NPJ Comput. Mater.* **10**, 254 (2024).

52. G. Kresse, J. Furthmüller, Efficient iterative schemes for ab initio total-energy calculations using a plane-wave basis set. *Phys. Rev. B Condens. Matter.* **54**, 11169–11186 (1996).

53. J. P. Perdew, K. Burke, M. Ernzerhof, Generalized gradient approximation made simple. *Phys. Rev. Lett.* **77**, 3865–3868 (1996).

54. P. E. Blöchl, Projector augmented-wave method. *Phys. Rev. B* **50**, 17953–17979 (1994).

55. K. Momma, F. Izumi, VESTA 3 for three-dimensional visualization of crystal, volumetric and morphology data. *J. Appl. Cryst.* **44**, 1272–1276 (2011).

56. A. Stukowski, Visualization and analysis of atomistic simulation data with OVITO—The Open Visualization Tool. *Model. Simul. Mater. Sci. Eng.* **18**, 015012 (2010).

57. R. Ram, L. Xia, H. Benzidi, A. Guha, V. Golovanova, A. G. Manjón, D. L. Rauret, P. S. Berman, M. Dimitropoulos, B. Mundet, E. Pastor, V. Celorrio, C. A. Mesa, A. M. Das, A. Pinilla-Sánchez, S. Giménez, J. Arbiol, N. López, F. P. G. de Arquer, Water-hydroxide trapping

in cobalt tungstate for proton exchange membrane water electrolysis. *Science* **384**, 1373–1380 (2024).

58. K. Yue, R. Lu, M. Gao, F. Song, Y. Dai, C. Xia, B. Mei, H. Dong, R. Qi, D. Zhang, J. Zhang, Z. Wang, F. Huang, B. Y. Xia, Y. Yan, Polyoxometalated metal-organic framework superstructure for stable water oxidation. *Science* **388**, 430–436 (2025).
